# Interplay and Targetome of the Two Conserved Cyanobacterial sRNAs Yfr1 and Yfr2 in *Prochlorococcus* MED4

**DOI:** 10.1038/s41598-019-49881-9

**Published:** 2019-10-04

**Authors:** S. Joke Lambrecht, Yu Kanesaki, Janina Fuss, Bruno Huettel, Richard Reinhardt, Claudia Steglich

**Affiliations:** 1 University of Freiburg, Faculty of Biology, D-79104 Freiburg, Germany; 2 NODAI Genome Research Center, Tokyo University of Agriculture, 1-1-1 Sakuragaoka, Setagaya-ku, Tokyo 156-8502 Japan; 3 Present Address: Research Institute of Green Science and Technology, Shizuoka University, 836 Ohya, Suruga-ku, Shizuoka 422-8529 Japan; 4 Max Planck-Genome-Centre Cologne, Max Planck Institute for Plant Breeding Research, D-50829 Köln, Germany

**Keywords:** Small RNAs, Marine biology

## Abstract

The sRNA Yfr1 and members of the Yfr2 sRNA family are almost universally present within cyanobacteria. The conserved motifs of these sRNAs are nearly complementary to each other, suggesting their ability to participate in crosstalk. The conserved motif of Yfr1 is shared by members of the Yfr10 sRNA family, members of which are otherwise less conserved in sequence, structure, and synteny compared to Yfr1. The different structural properties enable the discrimination of unique targets of Yfr1 and Yfr10. Unlike most studied regulatory sRNAs, Yfr1 gene expression only slightly changes under the tested stress conditions and is present at high levels at all times. In contrast, cellular levels of Yfr10 increase during the course of acclimation to darkness, and levels of Yfr2 increase when cells are shifted to high light or nitrogen limitation conditions. In this study, we investigated the targetomes of Yfr2, Yfr1, and Yfr10 in *Prochlorococcus* MED4, establishing CRAFD-Seq as a new method for identifying direct targets of these sRNAs that is applicable to all bacteria, including those that are not amenable to genetic modification. The results suggest that these sRNAs are integrated within a regulatory network of unprecedented complexity in the adjustment of carbon and nitrogen-related primary metabolism.

## Introduction

Small non-coding RNAs (sRNAs) are present within all domains of life and modulate the expression of target genes^1^. Compared to other regulators of gene expression, such as transcription factors, sRNAs exhibit some unique features with respect to regulatory dynamics, i.e., regulation by an sRNA leads to a faster decrease in the level of the protein product of the target gene than does regulation by a transcription factor^2^. Studies of sRNAs in bacteria and archaea show that they can regulate gene expression at the post-transcriptional level^1^, either by occluding or exposing ribosomal binding sites or ribonuclease cleavage sites^1^. Other distinct modes of sRNA action have been described that often require RNA chaperones such as Hfq or ProQ for their full functionality^1,3,4^.

In the most abundant photosynthetic organism on Earth^5^, the marine picocyanobacterium *Prochlorococcus*, sRNAs have been investigated since 2005^6^ and until today more than twenty were discovered in the model strain *Prochlorococcus marinus* MED4 (*Prochlorococcus* MED4)^7,8^. In contrast to the reduction in the number of protein regulators associated with the genome streamlining observed in this clade, the number of sRNAs is not reduced in *Prochlorococcus* MED4^7^. The relatively high number of sRNAs present in this bacterium may represent an adaptation to the extremely oligotrophic environment it inhabits, where nutrients are scarce and the synthesis of an RNA regulator requires fewer resources than that of a protein regulator^7^. Of the completely sequenced *Prochlorococcus* genomes, those of the low-light II/III ecotype are the only ones that do not contain *yfr1* genes encoding cyanobacterial functional RNA 1 (Yfr1), which are otherwise ubiquitous in cyanobacteria^9^. Most *yfr1* homologs are located between the genes encoding inosine-5′-monophosphate dehydrogenase (*guaB*) and thioredoxin (*trxA*)^10^. To date, only two porins, which originated from a gene duplication event, have been shown to be targets of Yfr1 in *Prochlorococcus* MED4^11^. In addition to *yfr1*, the *yfr2* sRNA family is similarly conserved and is present in all analysed cyanobacteria^12^. Unlike *yfr1*, *yfr2* genes are present in multiple copies in cyanobacterial genomes and occur at unconserved *loci*^12^. Many *Prochlorococcus* strains possess two copies of *yfr2*, although four gene copies are present in *Prochlorococcus* MED4 (*yfr2-5*) and *Synechococcus* CC9611 harbours nine^12^. With the exception of tmRNA, the 6 S and 4.5 S sRNAs and RNase P RNA, which are universally conserved throughout all bacterial phyla, all other sRNAs identified in *Prochlorococcus* are restricted to the marine picocyanobacteria (*Prochlorococcus* and marine *Synechococcus*) and do not occur outside this group^7,8^.

While it appears that *Prochlorococcus* relies heavily on sRNAs to modulate gene expression, the transcription of sRNAs is also coordinated by sigma factors and transcription factors. Despite the reduced set of regulatory proteins harboured by *Prochlorococcus* MED4, this bacterium encodes representatives of a variety of regulatory proteins. To activate regulatory pathways, *Prochlorococcus* MED4 encodes five sigma factors from groups 1 and 2, two Fur-type transcriptional regulators, three Crp-type transcriptional regulators (including NtcA), the LysR-type transcriptional regulator RbcR, the global response regulator LexA, one ArsR-type transcriptional regulator, two AbrB-type transcriptional regulators, the GntR-type transcriptional regulator PMM1637, and six two component response regulators (including RpaA, RpaB, and PhoB)^9^.

Studies of sRNA targets have developed from the prediction and verification of one or a few targets towards sequencing of the entire targetome of an sRNA, for which various approaches have been developed. RNA chaperones can be immunoprecipitated to allow for subsequent sequencing of the bound RNA (RIP-Seq)^13^. MAPS technology is based on the affinity purification of MS2-tagged sRNA and its bound targets followed by RNA-Seq, and this method can also be used to study sRNAs that function without an RNA chaperone^14^. Another approach, called GRIL-Seq, is based on the recombinant expression of T4 ligase and the sRNA of interest, taking advantage of proximity ligation and the subsequent enrichment of hybrid sRNA-target fragments followed by sequencing^15^. Target-enrichment sequencing of sRNAs (TEsR) uses a biotinylated *in vitro* transcript of an sRNA of interest to enrich targets that were reverse transcribed into cDNA^16^.

In this study, we established a new method for sRNA target enrichment that uses a hybrid sRNA-target pulldown approach we call CRAFD-Seq (cell-free RNA affinity pull-down followed by sequencing), a technique that is also applicable to organisms for which no genetic tools are available. Our CRAFD-Seq results suggest that Yfr2 interacts with both Yfr1 and the highly conserved sRNA Yfr10, which is less conserved in position and structure but contains the same conserved motif as Yfr1. Although Yfr1 and Yfr10 share a set of targets, these sRNAs also appear to have unique targets. The distinct targetomes of Yfr2, Yfr1 and Yfr10 coupled with their interdependency constitute a complex regulatory network comprising hundreds of genes in the *Prochlorococcus* MED4 genome.

## Results

### Use of CRAFD-Seq to identify the Yfr2 targetome

We recently showed that the GntR family transcriptional regulator PMM1637, which is common to all cyanobacteria, binds to promotor regions of *yfr2* homologs that contain the marine picocyanobacterial CGRE1 motif^17^. Because the CGRE1 motif is not present anywhere else in the *Prochlorococcus* MED4 genome, we investigated the regulon of Yfr2, resulting in an elucidation of the extended regulon of PMM1637.

Because tools for the genetic manipulation of *Prochlorococcus* do not currently exist, we developed a pipeline for the genome-wide enrichment of sRNA targets that is based on coupling *in vitro*-synthesized bait RNA to magnetic beads that are then incubated with total cell lysate (Fig. S1). Subsequently, a strand-specific cDNA library of specifically enriched target RNAs is prepared and amplified by PCR^18^ followed by sequencing. To identify enriched peaks, we developed a peak caller (Fig. S2) that is available to be downloaded (refer to the Material and Methods section for more details). We termed that method CRAFD-Seq for cell-free RNA affinity pull-down followed by sequencing. The advantage of this approach is that the targetome of any sRNA in any bacterium can be analysed, even in organisms such as *Prochlorococcus* that are recalcitrant to genetic manipulation.

**Figure 1:**
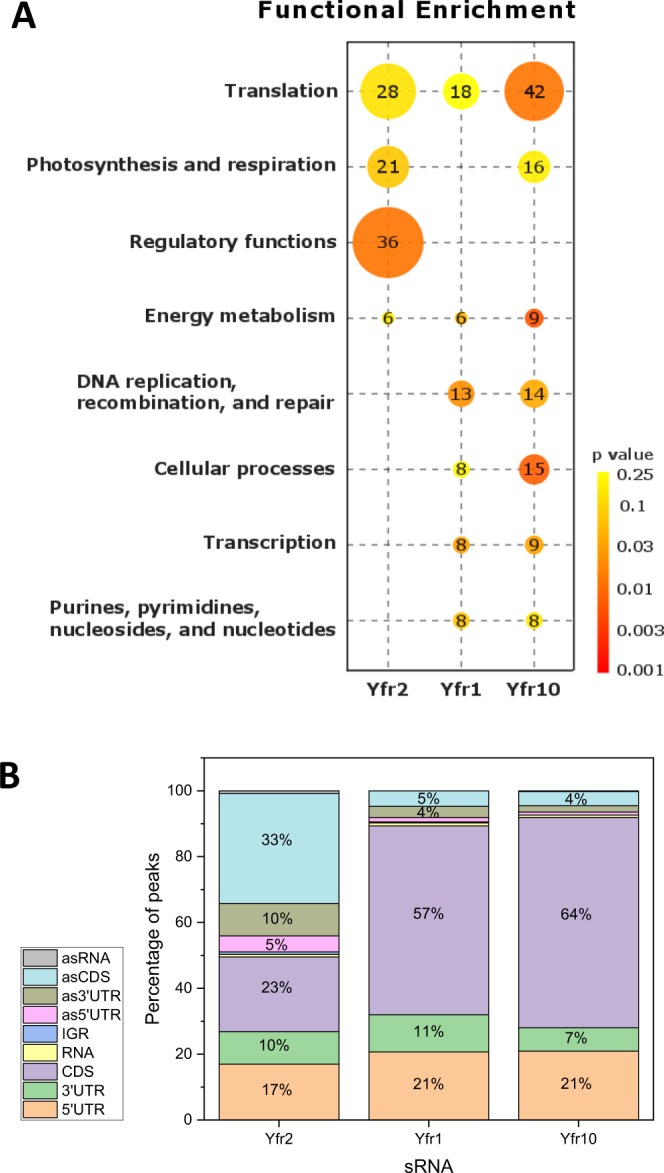
Overview on biological processes and sRNA-target RNA interaction regions regulated by Yfr2, Yfr1 or Yfr10. (**A**) Functional enrichment analysis based on CyanoBase functional categories. Only functional classes with a p-value of < 0.05 in at least one of the RNA affinity pull-down libraries are shown. (**B**) Relative distribution of enriched peaks located in gene coding regions (CDS), 5′ and 3′ untranslated regions (5′UTR and 3′UTR), untranslated genes (RNA) or antisense to CDS (asCDS), 5′/3′UTR (as5′UTR and as3′UTR) or RNA (asRNA).

We applied the CRAFD-Seq method using Yfr2 as bait with *Prochlorococcus* cells cultivated under standard growth conditions and identified 404 enriched peaks that could be assigned to 302 mRNAs (Table S1). For the mRNAs that bound on Yfr2, we observed a functional enrichment in the categories of translation, photosynthesis and respiration, regulatory functions and energy metabolism (Fig. 1A). Overall, the enriched peaks for the Yfr2 affinity pull-down assay were primarily located at sites that were antisense to a CDS (33%), inside of a CDS (23%), or within a 5′ UTR (17%) (Fig. 1B).

**Figure 2:**
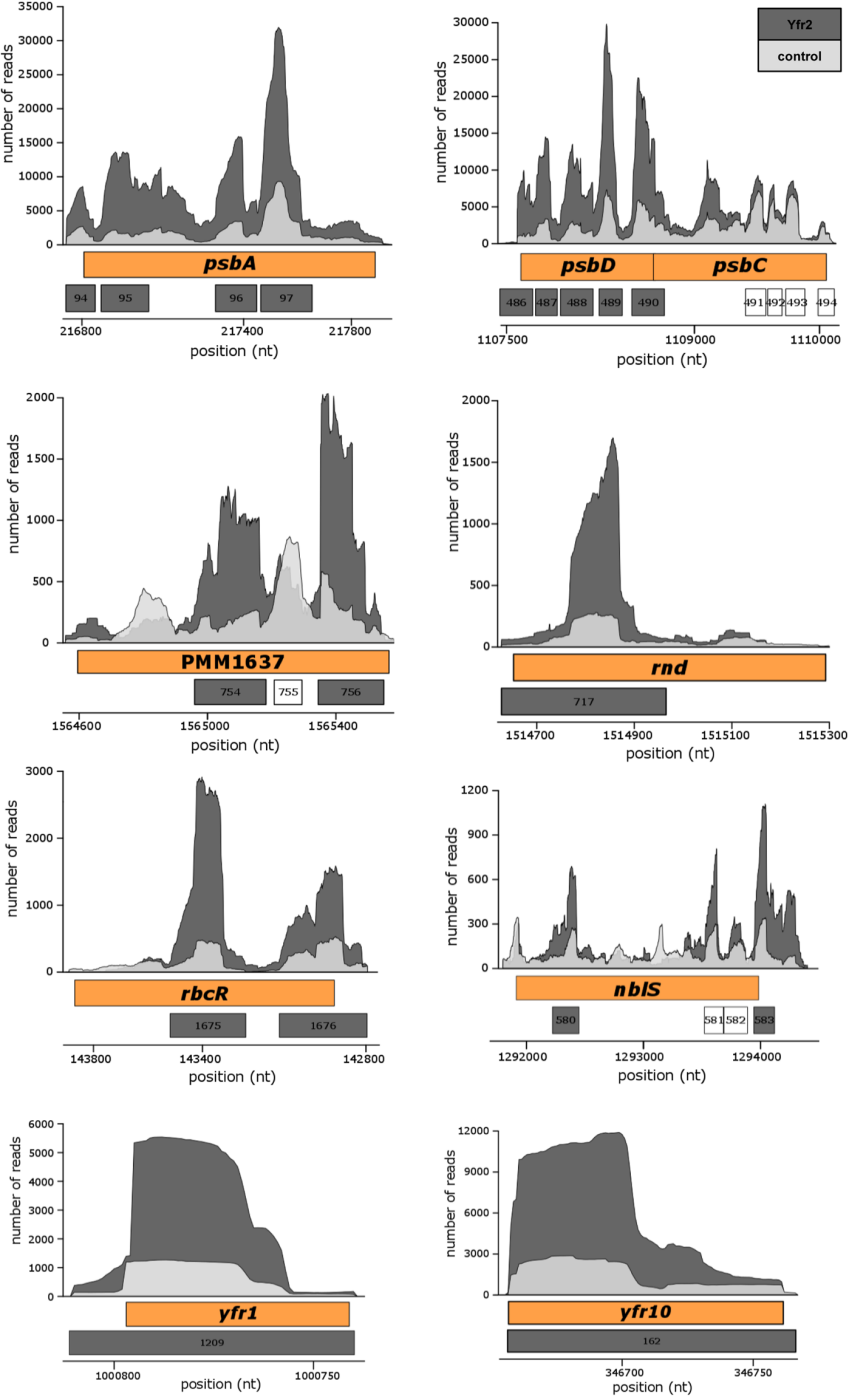
Coverage plots of selected Yfr2 targets discovered using the CRAFD-Seq approach. Mapped read regions of the Yfr2 affinity pull-down and the control libraries are coloured in dark and light grey, respectively. The orange boxes correspond to the gene positions of *psbA* and *psbC* (encoding the PSII reaction centre proteins D1 and D2, respectively), *psbD* (encoding the PSII core antenna protein CP43), PMM1637 (encoding the transcription factor GntR), *rnd* (encoding ribonuclease D), *rbcR* (encoding the Rubisco transcriptional regulator), *nblS* (encoding a two-component sensor histidine kinase) and the sRNAs *yfr1* and *yfr10*. The numbers in the grey boxes correspond to peak IDs of the Yfr2 enrichment library listed in Table S1. White boxes correspond to called peak regions that were not enriched. Genome coordinates for genes located on the reverse strand (*rbcR* and *yfr1*) are displayed with respect to the forward strand.

Among the genes, those belonging to the category photosynthesis and respiration were essential genes of PSII, such as *psbA*, which encodes the PSII reaction centre protein D1, and *psbD*, which encodes the PSII core antenna protein CP43 (Fig. 2). The enrichment of targets for regulatory functions expands the Yfr2 regulatory network even further. For example, the transcriptional regulator RbcR, which is believed to control genes of the Calvin-Benson-Bassham cycle and RubisCO^19^, as well as the two-component sensor histidine kinase NblS, which controls acclimation to high light via the response regulator RpaB^20^, were enriched targets of Yfr2 (Fig. 2). Intriguingly, we also observed enrichment of the GntR family transcriptional regulator PMM1637, which is a regulator of Yfr2 (Fig. 2). Furthermore, we detected ribonuclease D (encoded by *rnd*), which is responsible for the 3′ processing of tRNAs, in the Yfr2 dataset (Fig. 2). The mRNA of the *E*. *coli rnd* homologue is undetectable during stationary phase^21^, a time during which nutrients such as nitrogen are limiting. The induction of Yfr2 under nitrogen starvation may also lead to decreased levels of *Prochlorococcus* MED4 *rnd* mRNA, which would lead to an increase in its substrates during nitrogen deprivation.

### Yfr2 interferes with the interactions between Yfr1 and Yfr10 and their targets and is controlled by RNase E

**Figure 3:**
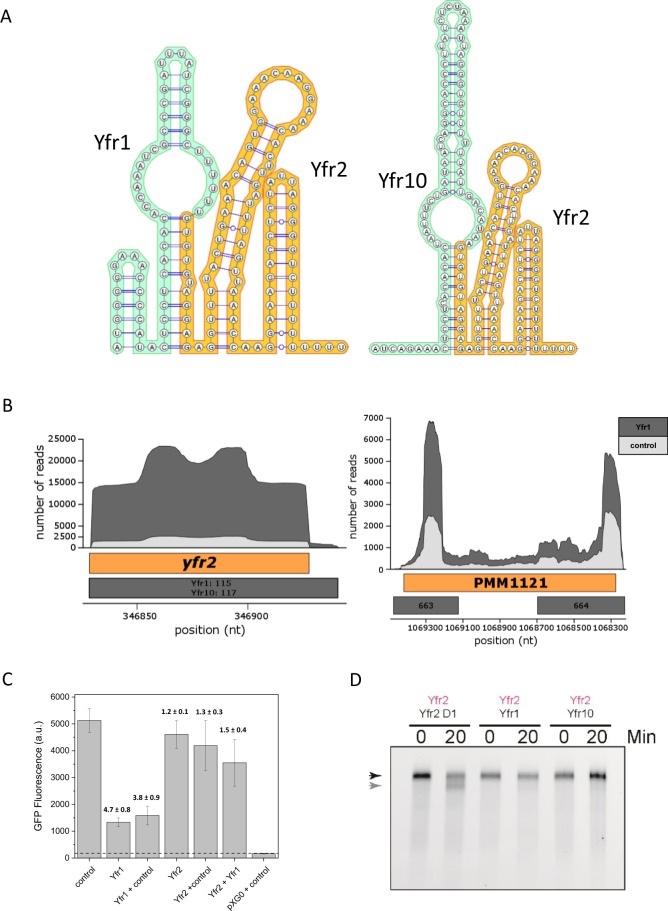
Effectors of Yfr2. (**A**) Co-folded structures of Yfr1-Yfr2 and Yfr10-Yfr2. (**B**) Read coverage plots of the Yfr1-enriched (dark grey) and the control library (light grey). The positions of genes are indicated by the orange boxes, and the numbers in the grey boxes correspond to peak IDs for the Yfr1 and Yfr10 enrichment libraries listed in Tables S2 and S3, respectively. The genome coordinates of PMM1121 are displayed with respect to the forward strand. (**C**) Verification of Yfr1-Yfr2 interaction by GFP reporter assay. Average florescence values were calculated for 50,000 events (cells), and the fold repression and respective error were calculated from twelve independent clones for each strain. Each transformed *E*. *coli* strain contains a plasmid carrying *sfgfp* fused to the PMM1121 5′UTR and a second plasmid expressing Yfr1 or Yfr2 alone or in combination with each other or a non-sense RNA (control). pXG0+ control does not express GFP and serves as negative control. The horizontal dashed line indicates background fluorescence. (**D**) Cleavage of Cy3-labelled Yfr2 (indicated by pink coloured text) by RNase E in the presence of equal amounts of unlabelled Yfr2 D1, Yfr1, or Yfr10. The black arrow indicates the full-length Yfr2 transcript and the grey arrow indicates the Yfr2 cleavage product.

Interestingly, two well-known sRNAs, Yfr1 and Yfr10, were also enriched in the Yfr2 affinity purification library (Table S1, Fig. 2). This result validates the earlier presumption of a physical interaction between the complementary regions of Yfr2 and Yfr1/10^7^ (Fig. 3A). In a previous study, we demonstrated the interaction of Yfr1 with two of the top *in silico*-predicted targets, the homologs PMM1119 and PMM1121^11^. To evaluate the ability of Yfr2 to interfere with interactions between Yfr1 and its targets, we modified a heterologous GFP reporter system to evaluate sRNA-mRNA interactions in *E*. *coli*^22^ by inserting both the sRNA genes *yfr1* and *yfr2*, each under the control of their own P_LlacO_ promoter, in the sRNA plasmid (for more details see the Materials & Methods section). The sRNA plasmids carrying *yfr1*, *yfr2*, *yfr1* + *yfr2*, a non-interacting control RNA (control) or *yfr1*/*yfr2*+ control were cotransformed with the plasmid carrying the PMM1121 5′ UTR fused to *sfgfp*. In the presence of Yfr1 or Yfr1+ control, a pronounced repression in GFP fluorescence (4.7-fold ±0.8 and 3.8-fold ±0.9, respectively) was observed (Fig. 3C). GFP fluorescence was restored to the initial levels when both the Yfr1 and Yfr2 sRNAs were coexpressed, demonstrating that Yfr2 counteracts the inhibition of translation initiation in the PMM1121 5′UTR caused by Yfr1 in *E*. *coli* (Fig. 3C).

Because endoribonuclease E (RNase E) frequently plays a role in sRNA-target interactions in bacteria^23^, we characterized the interaction between Yfr2 with Yfr1 or Yfr10 through RNase E *in vitro* cleavage assays. The results showed that Yfr2 is cleaved by RNase E and that this cleavage is abolished when Yfr2 binds to Yfr1 or Yfr10 (Fig. 3D). To exclude the impact of titration effects, we performed the RNase E cleavage assay in the presence of Yfr2 D1, an Yfr2 mutant missing the RNase E recognition site and the Yfr1 interaction region (Fig. S3). Cleavage assays conducted with Yfr1, Yfr10 or Yfr2 D1 alone *in vitro* showed that none of these RNAs is a substrate of RNase E (Fig. S3).

### Yfr1 and Yfr10 have overlapping but distinct target sets

Because of the unique interaction between the sRNAs Yfr2 and Yfr1 or Yfr10, we also used the CRAFD-Seq protocol to elucidate the targetomes of Yfr1 and Yfr10 by using these sRNAs as bait. The reciprocal fishing approach, using Yfr1 or Yfr10 as bait, largely confirmed that Yfr2 and its three homologs interact with Yfr1 or Yfr10 (Fig. 3B, Tables S2 and S3). For unknown reasons, Yfr2 was only slightly enriched in the Yfr10 library, but enrichment for the remaining Yfr2 homologs Yfr3, Yfr4, and Yfr5 was clearly above the set threshold. In total, we identified 288 and 384 enriched peaks associated with 206 and 253 mRNAs for Yfr1 and Yfr10, respectively.

Overall, the majority of peaks were located within a CDS (57 and 64% for Yfr1 and Yfr10, respectively), in a 5′ UTR (21% for Yfr1 and Yfr10), or in a 3′ UTR (11 and 7% for Yfr1 and Yfr10, respectively) (Fig. 1B). The functional enrichment of Yfr1 and Yfr10 targets was comparable, except for the photosynthesis- and respiration-associated targets, which were only enriched for Yfr10 (Fig. 1A). We compared the rank of the best intaRNA^11^-predicted interaction site for full-length genes of Yfr1, Yfr2 and Yfr10 with the lists of enriched/non-enriched CRAFD-Seq identified peaks (Tables S1–3, Fig. S4) and performed a non-parametric Kruskal-Wallis ANOVA test to show that intaRNA ranks are significantly better for enriched compared to non-enriched peaks with p-values of 1.69 × 10^−3^ (Yfr1), 1.74 × 10^−5^ (Yfr10) and 2.73 × 10^−7^ (Yfr2) (Fig. S4).

**Figure 4:**
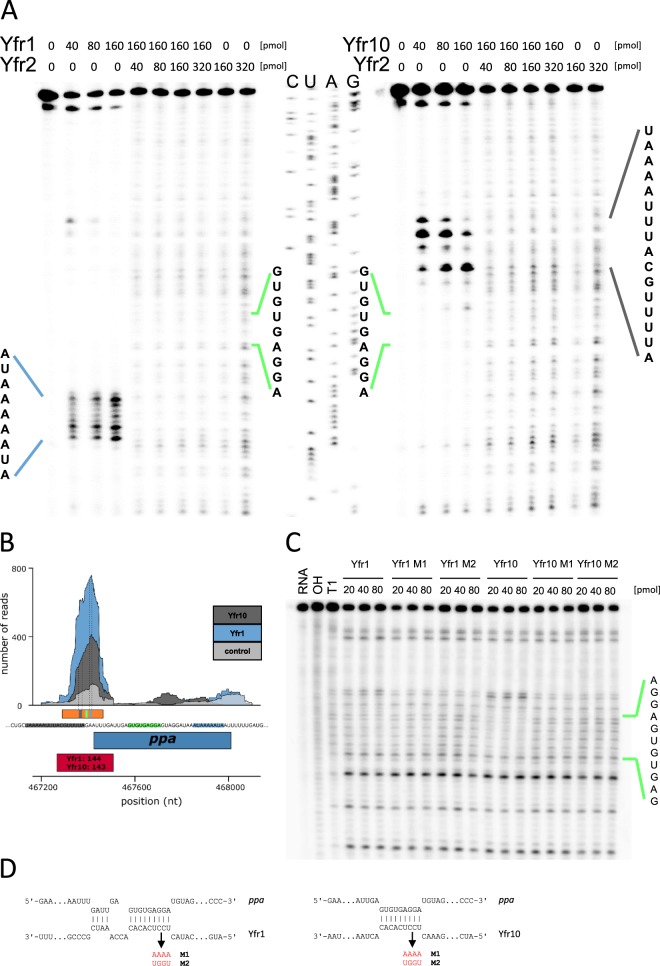
Yfr2 circumvents the interaction of Yfr1 and Yfr10 with their shared target inorganic pyrophosphatase (*ppa*). (**A**) Primer extension of 0.2 pmol *in vitro*-synthesized 5′ region *ppa* mRNA in the absence of any synthesized sRNA or in the presence of varying amounts of Yfr1 (left panel) or Yfr10 (right panel) alone or with Yfr1 (left panel) or Yfr10 (right panel) combined with Yfr2. Primer extension termination signals of *ppa* in presence of Yfr1 or Yfr10 are denoted in blue and grey lines, respectively. The interaction region predicted by intaRNA^11^ is shown in green lines. (**B**) Read coverage plots of the Yfr10- (dark grey), Yfr1-enriched (blue) and the control library (light grey). The position of *ppa* is indicated by the blue box, and the numbers in red boxes correspond to the peak IDs of the Yfr1 and Yfr10 enrichment libraries shown in Tables S2 and S3, respectively. The orange box marks the region of template DNA used for primer extension. The blue and grey boxes within the orange box correspond to the observed termination sites, and the green box corresponds to the predicted interaction site in A. The vertical dashed lines mark the start and end of the termination sites. The sequence of the 5′ UTR with Yfr1 (blue) and Yfr10 (grey) primer extension termination sites, the predicted interaction site (green) and the first ATG codon of the ORF are shown. (**C**) RNA footprinting assays of 0.1 pmol *in vitro*-synthesized 5′ region *ppa* mRNA in the presence of increasing amounts of Yfr1, Yfr1 M1, Yfr1 M2, Yfr10, Yfr10 M1 or Yfr10 M2, respectively. The first three lanes correspond to RNA – untreated control, OH – alkaline RNA ladder and T1 – RNase T1 treated *ppa in vitro* transcript. (**D**) Interactions between *ppa* and Yfr1/Yfr10 predicted by intaRNA^11^. The arrows indicate mutations M1 and M2 (red letters).

**Figure 5:**
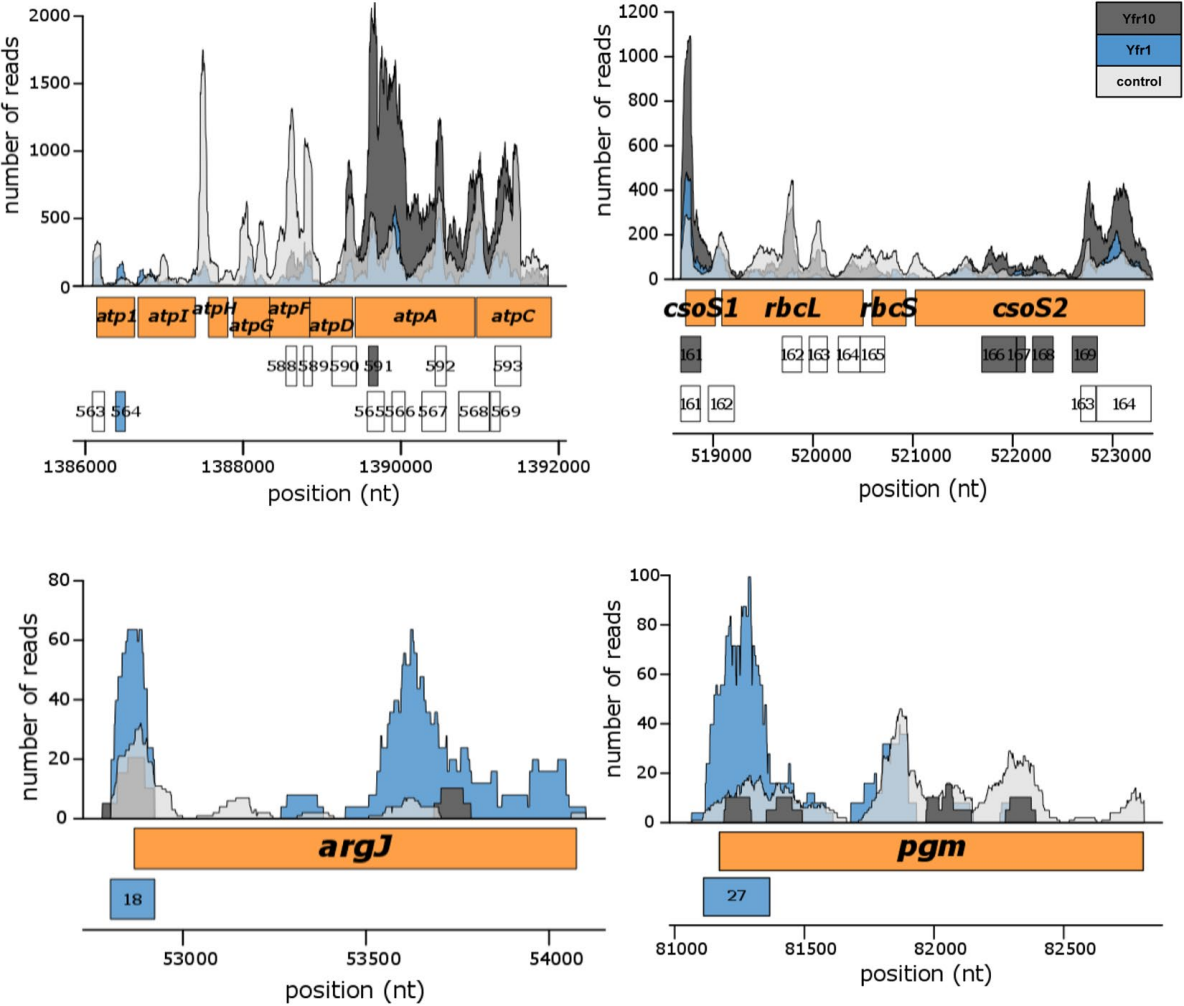
Coverage plots of selected Yfr1 and Yfr10 targets discovered using the CRAFD-Seq approach. Mapped read regions of the Yfr1 and Yfr10 affinity pull-down libraries are coloured in blue and dark grey, respectively, and those of the control library are shown in light grey. The orange boxes correspond to gene positions of parts of the ATP synthase operon, the carboxysome shell polypeptides encoded by *csoS1* and *csoS2*, the small and large subunits of Rubisco (encoded by *rbcS* and *rbcL*, respectively), *argJ* (encoding a bifunctional ornithine acetyltransferase/N-acetylglutamate synthetase) and *pgm* (encoding phosphoglucomutase). The numbers in the grey or blue boxes correspond to the peak IDs of Yfr1 and Yfr10, respectively, listed in Tables S2 and S3, respectively. White boxes correspond to called peak regions that were not enriched.

The CRAFD-Seq results also verified that the mRNA of PMM1121 is a target of Yfr1 (Fig. 3B) as well as those of the previously suggested Yfr1 targets^11^ PMM1119 (the homolog of PMM1121), inorganic pyrophosphatase (*ppa*, PMM0494) and the bifunctional ornithine acetyltransferase/N-acetylglutamate synthetase (*argJ*, PMM0050) (Table S2). The latter two targets could not be previously verified using the GFP reporter assay because of the poor translation efficiency of their UTRs in *E*. *coli*^11^. Inorganic pyrophosphatase plays an important role in the oxidative phosphorylation pathway by splitting pyrophosphate into two molecules of inorganic phosphate that can subsequently be added to ADP by the ATP synthase complex. The peak areas located in the 5′ region of *ppa* (Fig. 4B) were 6- and 3-fold enriched in Yfr1 and Yfr10 pulldown libraries, respectively (Tables S2 and S3). We further validated these interactions by primer extension and showed that the termination signals of *ppa* vary between Yfr1 and Yfr10, which might be explained by different structures that are formed during the interaction of Yfr1-*ppa* and Yfr10-*ppa* complexes, respectively (Fig. 4A). However, our data indicate that the interaction sites of both Yfr1 and Yfr10 with *ppa* are at the same position, the same position that was predicted by intaRNA^11^ (Fig. 4C,D). The formation of RNA-RNA complexes is stronger for *ppa*-Yfr10 than for *ppa*-Yfr1, which might be explained by structural differences in the complexes that are also suggested by primer extension results. We exchanged UCCU of the Yfr1/Yfr10 conserved motif with AAAA (M1) or UGGU (M2) (Fig. 4D). Neither of the mutants were able to interact with *ppa* as indicated by the loss of the footprint (Fig. 4C). The affinity of the sRNAs Yfr1 or Yfr10 to Yfr2 is stronger compared to the mRNA target *ppa* as the interaction of Yfr1 or Yfr10 with *ppa* was completely inhibited by Yfr2, even when the latter sRNA was present in very small amounts (Fig. 4A). Despite the identical conserved motifs in Yfr1 and Yfr10, we observed distinct sets of enriched mRNAs for the Yfr1 and Yfr10 targetomes. Almost half of all the Yfr1 targets were not present in the Yfr10 library, and two thirds of the targets were unique to Yfr10 (Fig. S5). In contrast to *ppa*, *argJ* and phosphoglucomutase (*pgm*, PMM0076) are targeted by Yfr1 but not Yfr10 (Fig. 5). Amongst the specific targets of Yfr10 are *atpA* and *atpC*, which encode the α and γ subunits of the F_1_ region of ATP synthase, as well as *csoS1* and *csoS2*, which encode carboxysome shell polypeptides (Fig. 5). In both cases, the genes are organized within operons, and all of the other genes within these operons are not targeted by Yfr10 (Fig. 5).

### Complex post-transcriptional regulation of carbon primary metabolism

**Figure 6:**
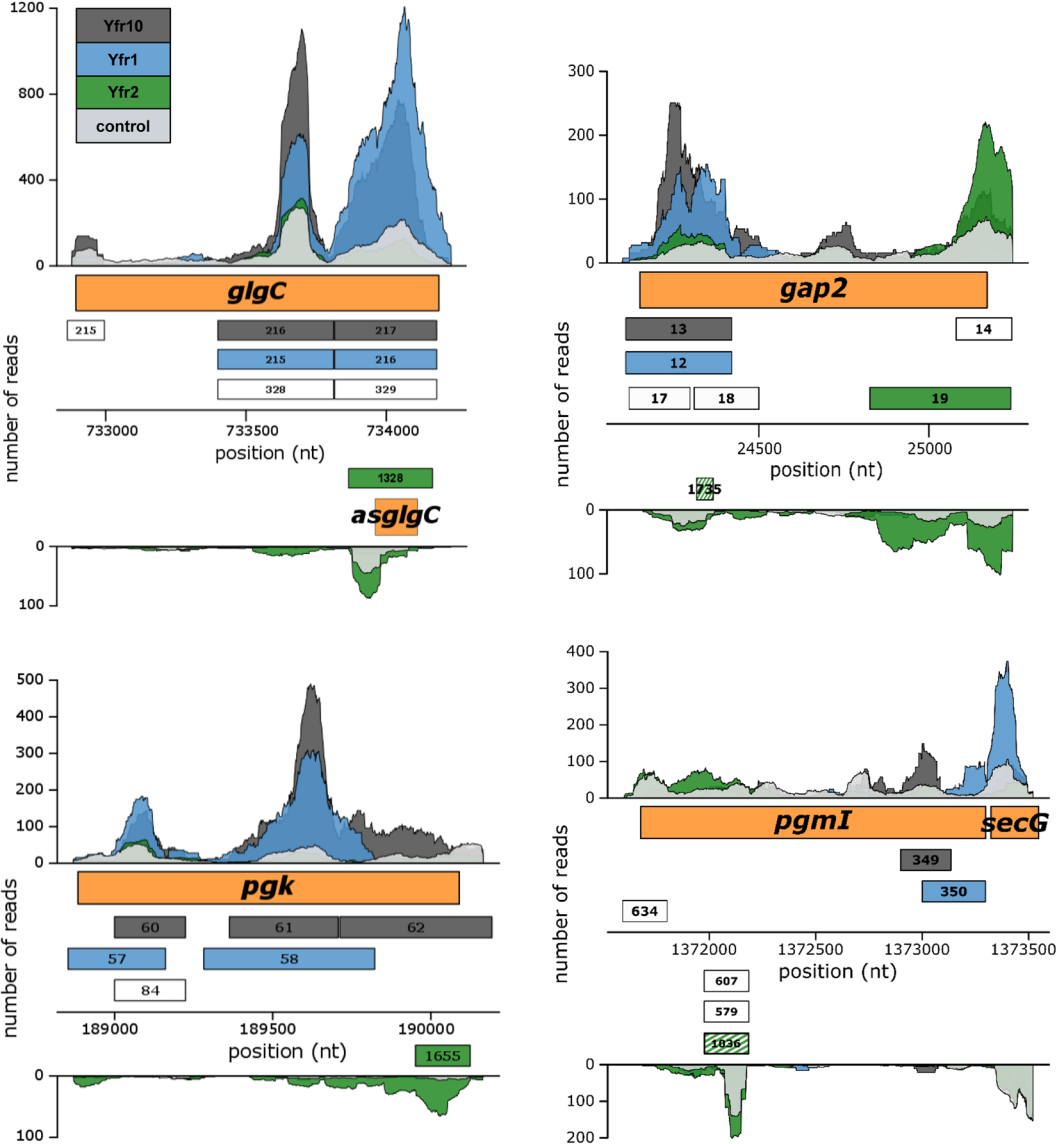
Coverage plots of selected Yfr2, Yfr1 and Yfr10 targets discovered using the CRAFD-Seq approach. Mapped read regions of the Yfr2, Yfr1 and Yfr10 affinity pull-down libraries are coloured in green, blue and dark grey, respectively, and those of the control library are shown in light grey. The orange boxes correspond to gene positions of *glgC* (encoding glucose-1-phosphate adenyltransferase) and *asglgC*, *gap2* (encoding glyceraldehyde-3-phosphate dehydrogenase), *pgk* (encoding phosphoglycerate kinase) and *pgmI* (encoding phosphoglycerate mutase). The numbers in the grey, blue and green boxes correspond to the peak IDs of Yfr10, Yfr1 and Yfr2 listed in Tables S1, S2 and S3, respectively. Boxes coloured in green and white stripes correspond to peak regions where only one of the duplicate libraries was more than 2-fold enriched. White boxes correspond to called peak regions that were not enriched.

We observed enrichment for several mRNAs encoding enzymes involved in carbon primary metabolism in both libraries of Yfr1 and Yfr10. In addition, these mRNAs possess asRNAs that were enriched in the Yfr2 library. The glucose-1-phosphate adenyltransferase (*glgC*, PMM0769) mRNA appears to be under the control of a highly complex regulatory network of non-coding RNAs, including Yfr1/10, Yfr2 and the *cis*-encoded antisense RNA (asRNA) asGlgC (Fig. 6). The *glgC* mRNA is internally targeted by Yfr1 and Yfr10 as well as by asGlgC (Fig. 6), which we first observed in a previous study^8^. Peak 1328 corresponds to asGlgC and was enriched 2.6-fold in the Yfr2 affinity-purified library (Table S1), indicating that asGlgC is regulated by Yfr2. A similarly complex regulatory circuit can be anticipated for *gap2* (glyceraldehyde-3-phosphate dehydrogenase, PMM0023), *pgk* (phosphoglycerate kinase, PMM0195) and *pgmI* (phosphoglycerate mutase, PMM1434), which appear to be controlled by Yfr1/Yfr10 and corresponding asRNAs, the latter of which seems to be under the control of Yfr2 (Fig. 6). Regulation of asRNAs by Yfr2 is a common feature, as almost half of all enriched peaks (48%) are located antisense to RNAs (peaks antisense to the CDS and the 5′ and 3′ UTRs combined), whereas this fraction makes up less than 10% for Yfr1 and Yfr10 (Fig. 1B).

### Differential expression and conservation of Yf1, Yfr2 and Yfr10

**Figure 7:**
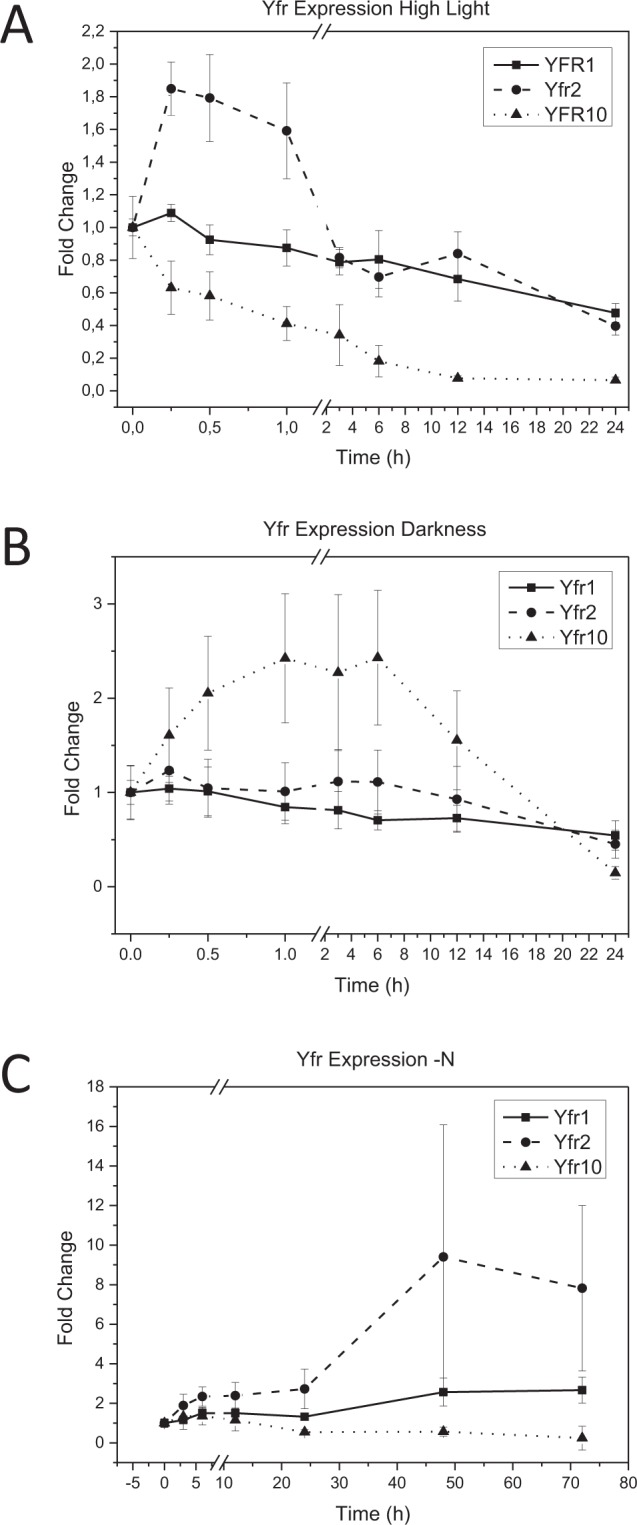
Temporal expression profiles of Yfr1, Yfr2 and Yfr10 during nitrogen depletion, high light-, or darkness stress. (**A**) *Prochlorococcus* MED4 cells were shifted from 30 µE to 300 µE, and samples were taken at 0, 0.25, 0.5, 1, 3, 6, 12, and 24 h after stress application and were probed against Yfr1 and Yfr10. (**B**) Acclimation of *Prochlorococcus* MED4 to darkness. Samples were taken at 0, 0.25, 0.5, 1, 3, 6, 12, and 24 h after stress application and were probed against Yfr1, Yfr2, and Yfr10. (**C**) Yfr1 and Yfr10 expression profiles of *Prochlorococcus* MED4 samples after 0, 3, 6, 12, 24, 48, and 72 h of nitrogen starvation. Data for Yfr2 during high light stress (**A**) and nitrogen starvation (**C**) were replotted from^17^. All stress experiments were carried out in biological triplicates. Extracted RNA samples were separated on 7 M urea-10% PAA gels, transferred on Hybond N+ membranes and hybridized with the respective probe.

To improve our understanding of the interaction between Yfr2 and Yfr1 or Yfr10 and of their distinct targetomes, we investigated the expression of Yfr2, Yfr1, and Yfr10. Yfr2 was previously shown to be induced during late nitrogen starvation and early high light stress^17^. The abundance of Yfr1 and especially Yfr10 declines during high light acclimation (Fig. 7A), prompting us to investigate the effect of darkness on Yfr2, Yfr1, and Yfr10 expression. While Yfr1 and Yfr2 are rather stable, Yfr10 was 2.4-fold upregulated after one hour of darkness (Fig. 7B). Yfr10 is especially downregulated during elongated nitrogen starvation, whereas Yfr1 is at least 2.6-fold induced after 48 h (Fig. 7C).

Until this study, it was assumed that Yfr1 and the Yfr2 sRNA family are present in almost all cyanobacteria, whereas Yfr10 appeared to be restricted to a few *Prochlorococcus* strains despite harbouring the same motif as Yfr1^7^. We showed that Yfr10-like sRNAs are more common than previously believed. For example, we verified the presence of Yfr10 in the marine picocyanobacterium *Synechococcus* WH8102 (Fig. S6). Furthermore, other species within the cyanobacterial phylum possess sRNAs that encode the conserved Yfr1 motif in addition to Yfr1, such as Syr5 in *Synechocystis* sp. PCC 6803^24^, TSS 2515196r and TSS 3750139 f in *Nostoc* sp. PCC 7120^25^ and Yfr104 in *Prochlorococcus* MIT9313^8^ (Fig. S7). Intriguingly, we also detected Yfr10 in *Prochlorococcus* SS120 (Fig. S6), which was previously considered to be devoid of a Yfr1 homolog^9^ but is now known to at least possess an sRNA with the Yfr1 motif.

## Discussion

We previously showed that two Yfr2 homologs in *Prochlorococcus* MED4 are regulated by the cyanobacterial GntR transcriptional regulator PMM1637^17^. Studies on homologous GntR proteins in other cyanobacteria, such as *Synechocystis* sp. PCC 6803, showed that GntR mutants fail to establish correct ratios of PSII/PSI during acclimation to high light^26^ and fail to degrade phycobilisomes during nitrogen starvation^27^. Consistent with these findings, we observed increased expression of Yfr2 during both conditions^17^. Our findings that Yfr2 possibly targets genes involved in photosynthesis and respiration are therefore in good agreement with the results of these previous studies. Changing light conditions and nitrogen availability are presumably the most important factors that cyanobacteria, especially *Prochlorococcus*, have to cope with in the oligotrophic oceans. Therefore, it is reasonable that Yfr2 also targets other regulatory factors, such as the transcriptional regulator *rbcR*, the sensor histidine kinase *nblS* or asRNAs, which together comprise 48% of the enriched peaks. Yfr2 thereby integrates the perceived multiple environmental signals into other regulatory circuits. Because we can assume that at least one function of antisense transcripts is the regulation of *cis*-encoded genes, asRNAs can be added to the regulatory functions category of protein-coding genes. Furthermore, the high number of enriched antisense peaks highlights the importance of antisense transcription for gene regulation. These findings emphasize the utility of Yfr2 as a modulator of gene expression of regulatory elements.

We previously showed that PMM1637 does not act as an autoregulator^17^. However, the results from this study indicate that a feedback loop occurs via Yfr2. Modulation of gene expression of an sRNA by a transcription factor has been previously described. For instance, the sRNAs NsiR4 in *Synechocystis* sp. PCC 6803 and NsrR1 in *Nostoc* sp. PCC 7120 are regulated by NtcA^28,29^. However, feedback control of an sRNA on its associated transcription factor has only been described in a few instances in bacteria. In *Vibrio harveyi*, the quorum-sensing related Qrr sRNAs posttranscriptionally repress LuxO, which functions as their transcriptional repressor^30^. Our data suggest the existence of feedback control of Yfr2 on its transcriptional regulator PMM1637, adding another example to this class of control mechanisms. We further showed that the activity of Yfr2 can be neutralized either by its pairing with Yfr1/10 or through its RNase E-mediated cleavage. It is unclear if the cleavage of Yfr2 by RNase E initiates its degradation. However, in addition to the generally very high stability of Yfr2^6^, the Yfr2 cleavage product possibly represents a modified version of the sRNA that is unable to interact with Yfr1 or Yfr10 via its conserved 5′ motif and can only facilitate gene regulation via the conserved motif in the first stem loop^12^.

The CRAFD-Seq results obtained in our study revealed a relatively high occurrence of enriched peaks within CDS regions. This result is in contrast to those obtained in RIL-Seq experiments, where an almost equal distribution of chimeric sRNA fragments with CDS and 5′ UTRs was observed^31^. These differences may be explained by differences in the experimental setup or a by a different mode of action of sRNAs in *Prochlorococcus*. The results of recent ribosomal profiling studies suggest that translational inhibition via blocking of the Shine-Dalgarno sequence (SD) is possibly not as established as previously thought for cyanobacterial sRNAs and their targets^32^. The SD sequence does not occur more often than by chance in *Prochlorococcus* MED4 and MIT9313^8^, and in *Synechocystis* sp. PCC 6803 it is only detected in 26% of all genes^33^. The latter number of SD sequences in *Synechocystis* sp. PCC 6803 correlates well with the 27% of genes that were observed to have a higher than average intergenic coverage in the ribosomal profiling experiment for which SD sequences were detected^32^. The genes containing SD sequences were enriched in the categories of photosynthesis and respiration and for translation, possibly because genes in these categories are highly translated^32^. However, this does not appear to be the case for *Prochlorococcus*, and further studies are required to examine the detailed influence of internally binding sRNAs in *Prochlorococcus* on their targets.

During nitrogen starvation, cells undergo chlorosis and reduce the levels of their photosynthetic apparatus, since phycobiliproteins and chlorophyll are rich in nitrogen that can be recycled. This process is abolished in mutants of the cyanobacterial GntR transcriptional regulator in *Synechocystis* sp. PCC 6803 despite the presence of elevated *nblA* levels^27^. Furthermore, nitrogen starvation is also indicative as an excess of carbon supply^34^. The metabolic fluxes of fixed carbon are therefore rerouted from amino acid synthesis, which cannot be sustained, towards gluconeogenesis^35,36^. The genes encoding enzymes that metabolize fixed carbon in the form of 3-phosphoglycerate, either in the direction of gluconeogenesis (such as *pgk* and *gap2*) or towards the citrate cycle (such as *pgmI*) are regulated by Yfr1 and/or Yfr10. However, because Yfr2 can interact with Yfr1/10, the stimulus of late nitrogen or high light stress can be integrated into these metabolic pathways via Yfr1/10 and through the Yfr2-controlled asRNAs of *pgk*, *gap2*, and *pgmI*. The pathway from 3-phosphoglycerate towards the citrate cycle appears to be indirectly controlled by Yfr2, because *pgmI* is also a target of Yfr2, possibly enhancing the shutdown of this route for fixed carbon. A highly complex regulatory circuit involving Yfr2, Yfr1/10 and *asglgC* can be similarly anticipated for *glgC*, which catalyses the first committed step in glycogen synthesis. Another target of Yfr1 is the important metabolic branch point enzyme phosphoglucomutase (encoded by *pgm*), which links glycolysis, the oxidative pentose phosphate pathway and glycogen metabolism, making it directly involved in the processes of polyglucan storage (glycogen synthesis) and utilization (e.g., respiration via the glycolysis and OPP pathways)^37^. Unfortunately, in this study we only determined the targetomes of Yfr1, Yfr2, and Yfr10, and a functional analysis of the mode of action of the assayed sRNAs requires further investigations.

Comparing the expression profiles of an sRNA and its targets may provide useful information on the mode of interaction. Previous studies showed that *ppa* expression is downregulated during high light stress^38^ and nitrogen depletion^39^, both conditions where Yfr2 is upregulated. This result may suggest that Yfr1 and Yfr10 have stabilizing effects on *ppa* mRNA. A similar observation was made for the Yfr10-specific targets of the carboxysome-associated genes *csoS1* and *csoS2* and for *atpA* and *atpC*, which are downregulated during nitrogen stress^39^. However, because multiple regulatory inputs affect one gene, and the mode of action for sRNAs in *Prochlorococcus* is enigmatic, we could not detect this effect in general for the sRNA targets presented here.

The importance of Yfr1, beside its interaction with targets involved in central pathways such as energy metabolism, is supported by its almost omnipresent occurrence in members of the cyanobacterial phylum^10^. This idea is further confirmed with the discovery of a second sRNA (Yfr10) that shares the same conserved motif as Yfr1^7^. Unlike Yfr1, synteny and sequence conservation, except from the Yfr1 motif, is not conserved, resulting in secondary structure conservation among Yfr10 homologs being very low (Fig. S7). These features could be the reason that the context of additionally encoded Yfr1 motifs remained undiscovered by computational methods. In HL-adapted *Prochlorococcus* strains, Yfr10 homologs are encoded upstream of Yfr2 homologs^17^. The proximity of expression of both sRNAs possibly enhances their interaction potential. Previous studies suggested that Yfr1 is required for growth during various stresses^40^ and may explain why *Prochlorococcus* SS120, which lacks Yfr1^9^, is especially susceptible to abiotic stresses. Thus, *Prochlorococcus* SS120 may only be viable because it still possesses Yfr10, which is able to partially fulfil the functions of Yfr1, a theory that is supported by the observed overlap between the Yfr1 and Yfr10 regulons (Fig. S5). However, we were surprised to observe that half of the enriched genes for Yfr1 and two thirds of those for Yfr10 were sRNA-specific (Fig. S5), which clearly shows that RNA-RNA interactions are very complex and cannot be limited to simple complementarity. The conserved motif in Yfr1 is a single-stranded region between two stem loops^10,40^, whereas the conserved motif in Yfr10 is present in the single-stranded loop on top of a stem^7^. The different accessibility and surroundings of the conserved motif appears to enable the discrimination of targets.

**Figure 8:**
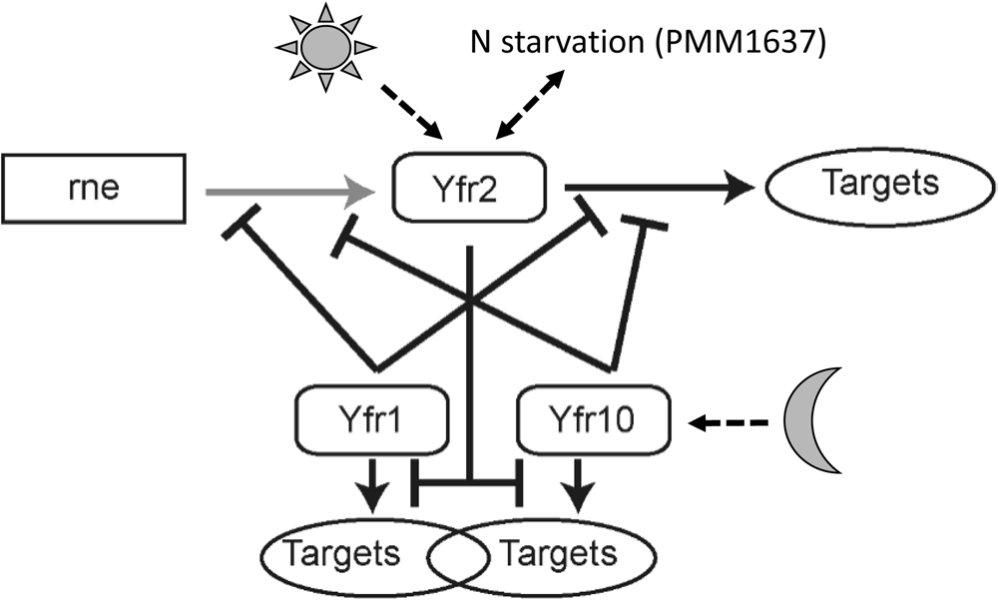
Schematic representation of the interplay between Yfr2, Yfr1, Yfr10, RNase E and their targets. Central to this model is the capability for titration between Yfr2 and Yf1/Yfr10 sRNAs. Therefore, changes in the concentration of any of these sRNAs will also affect the possible outcome by any of the other sRNAs. Interactions drawn in black represent RNA-RNA interactions and those in grey represent RNA-protein interactions. Arrowheads on the tips indicate an unknown mode of action, while bars on the tips indicate inhibition. Dashed black arrows indicate abiotic factors that modulate the gene expression of Yfr2 and Yfr10.

Collectively, our data suggest the existence of a complex, interconnected network of interactions among Yfr2, Yfr1 and Yfr10 and their targets (Fig. 8). We identified late nitrogen starvation and high light stress as important factors that trigger the induction of Yfr2 mediated by the GntR transcription factor PMM1637. A change in the light regime (high light and darkness) regulates the expression of Yfr10, although the regulators involved in the modulation of *yfr10* gene expression during varying light conditions are unknown. However, *yfr1* is constitutively expressed under all stress conditions assayed in this study. In addition to the reciprocal influence of Yfr2 and Yfr1/10, the cleavage of Yfr2 by RNase E is another layer of regulation in this system that impedes the interaction between Yfr2 and Yfr1/10 and presumably of Yfr2 with many of its targets.

## Materials and Methods

### Culturing and RNA preparation

*Prochlorococcus* MED4 cultures were grown at 22 °C in AMP1 medium^41^ under 30 µmol quanta m^−2^ s^−1^ of continuous white cool light to cell densities of 1–3 × 10^8^ cells per ml. Stress experiments for high-light and nitrogen starvation were performed as described previously^17^. For darkness experiments, cells were transferred from 30 µmol quanta m^−2^ s^−1^ of continuous white cool light to complete darkness for 0, 15, or 30 min or for 1, 3, 6, 12, or 24 h, with the cells subsequently harvested in darkness by filtration as previously described^17^. RNA extraction and northern hybridization were performed as described previously^6,17^. *Prochlorococcus* SS120 and *Synechococcus* sp. WH8102 cultures were grown at 22 °C in AMP1 medium^41^ under 15 µmol quanta m^−2^ s^−1^ of continuous white cool light, and nitrogen depletion experiments were performed as described preveiously^17^. Yfr10 homologs were probed with primers #1–3 (Table S4).

### *In vitro* RNA synthesis and biotinylation of RNA

Transcript templates for *in vitro* RNA synthesis were generated from purified PCR products or annealed complementary oligonucleotides using primers #4–13 (Table S4). The desired RNAs were transcribed using a MegaShort script Kit (ThermoFisher Scientific), and residual DNA was removed by TURBO DNase I treatment, with both steps performed according to the manufacturer’s instructions. RNA was purified and concentrated by phenol-chloroform extraction and ethanol precipitation or using RNA Clean & Concentrator columns (Zymo Research) following the manufacturer’s instructions. For the primer extension assay, if required, *in vitro*-transcribed RNA was separated on 7 M urea-10% polyacrylamide gels or on 2% non-denaturing agarose gels, and full length fragments were excised and purified using either a ZR small-RNA PAGE Recovery Kit (Zymo Research) or a NucleoSpin Gel and PCR-Clean-up kit (Macherey-Nagel) according to manufacturer’s instructions. For biotinylation, two volumes of KIO_4_ (6 mM) were added to 1.5 nmol of RNA and was incubated at room temperature, in the dark for one hour. Subsequently, one volume of ethylene glycol/H_2_O (1:1) was added, and the RNA was precipitated by adding 2.9 volumes of ethanol (100%) and 0.1 volumes of NaCl (3.3 M) at −20 °C for a maximum of 60 min. The RNA was centrifuged at 13,000 g for 30 min at 4 °C, the supernatant was removed, and the pellet was washed with 70% ethanol. The air-dried pellet was then resuspended in 24 µl 10 × PBS (pH 7.4), 6 µl H_2_O, and 20 µl EZ-Link Alkoxyamine-PEG12-Biotin (50 mM in DMSO). The solution was incubated at 37 °C in the dark for 3 h and was periodically mixed, after which 50 µl of NaBH_4_ (20 mM) and 100 µl of Tris-HCl (0.1 M, pH 7.5 at room temperature) were added and the mixture was incubated in the dark at 4 °C for 30 min. After the RNA was precipitated overnight as described above, free EZ-link-PEG12-alkoxyamine was removed by washing the RNA with water in Amicon ultracel 10-K columns (Merck).

### Affinity purification of target RNAs

*Prochlorococcus* MED4 cultures (500 ml for each sRNA to be analysed) were pelleted at 15,000 g for 10 min at 22 °C. The cell pellets were washed with a solution containing 1.3 M betaine and 10 mM HEPES (pH 8) and were centrifuged again. The pellets were resuspended in lysis buffer (100 mM Tris-HCl (pH 7.5), 500 mM LiCl, 10 mM EDTA, 5 mM DTT, and 1% LiDS) and lysed using a OneShot constant pressure system (Constant Systems Limited) at 1.5 kBar. Cell debris was separated from the cell lysate by centrifugation for 30 min, 13,000 g, at 4 °C. For immobilization of the bait RNA, 250 µl of Dynabeads Streptavidin MyOne C1 (ThermoFisher Scientific) per sample were prepared following the manufacturer’s instructions. The beads were washed once with binding buffer (20 mM Tris-HCl (pH 7.5), 1 M LiCl, and 2 mM EDTA). Biotinylated RNA was then loaded onto the beads in binding buffer for 10 min at room temperature with gentle agitation. Subsequently, the beads were washed first with binding buffer then with lysis buffer. To reduce the binding of nonspecific RNA to the beads, the cell lysate was first incubated with unloaded beads, which were prepared as described above, for 10 min at room temperature with gentle agitation. The unbound cell lysate incubated with the empty beads was then incubated with beads charged with the sRNA of interest for 10 min at room temperature with gentle agitation. The beads were washed with 1 ml buffer A (10 mM Tris-HCl (pH 7.5), 0.3 M LiCl, 1 mM EDTA, and 0.1% LiDS), 1 ml buffer B (10 mM Tris-HCl (pH 7.5), 0.3 M LiCl, and 1 mM EDTA), and 0.5 ml buffer C (10 mM Tris-HCl (pH 7.5), 0.15 M LiCl, and 1 mM MgCl_2_). Bound RNA was eluted in 150 µl of elution buffer (10 mM Tris-HCl, pH 7.5) by incubating the sample for 2 min at 65 °C, and the beads were immediately removed by placing the tube on a magnet and transferring the supernatant to a new tube.

### Primer extension

The primers were labelled as described previously^42^. Annealing mixtures containing 0.2 pmol of *in vitro*-synthesized target RNA, 2 pmol of the 5′ end-labelled primer ppa_RT (Table S4) without or with 40/80/160/320 pmol of *in vitro*-synthesized sRNAs (Yfr1, Yfr2, or Yfr10) were heated for 10 min at 70 °C and then chilled on ice for at least 5 min. cDNA synthesis was performed for 2 h at 30 °C using SuperScript III Reverse Transcriptase (ThermoFisher Scientific) according to the manufacturer’s instructions. The reaction was inactivated by incubation for 15 min at 70 °C, which was followed by RNase H treatment for 20 min at 37 °C and a final heat inactivation for 5 min at 95 °C in RNA loading buffer. DNA sequencing ladder reactions were performed with the same 5′ end-labelled primer used for cDNA synthesis and the same template DNA used for target RNA *in vitro* synthesis using a USB Thermo Sequenase Cycle Sequencing Kit (Affymetrix). Primer extension products and sequencing reactions were separated on 8.3 M urea-6% polyacrylamide sequencing gels, and the vacuum-dried gels were exposed to imaging plates. Signals were visualized using a Thyphoon FLA 9500 instrument (GE Healthcare) with Quantity One software (Bio-Rad).

### RNA footprinting

*In vitro*-synthesized RNA was produced from annealed primers #4, 5, 23–34 (Table S4) using the HighScribe T7 Quick High Yield RNA Synthesis Kit (NEB). For removal of 5′ triphosphates, 20 pmol *ppa in vitro*-synthesized RNA was incubated with 20 units FastAP (Thermo Fischer Scientific) in a 200 µl reaction volume at 37 °C for one hour. Dephosphorylated *in vitro*-synthesized RNA was labeled with [γ-^32^P] ATP by T4 Polynucleotide Kinase (Thermo Fischer Scientific). RNA footprinting was performed using Ambion RNase T1 according to manufacturer’s description. Briefly, 0.1 pmol of labeled *ppa in vitro*-synthesized RNA was mixed with 20, 40, or 80 pmol of unlabeled Yfr1, Yfr10, or mutant *in vitro*-synthesized RNA, denatured for 1 min at 95 °C and cooled to room temperature for 5 min. Subsequently, 1 µl of 1 µg/µl yeast RNA and 1 µl of 10 x structure buffer was added and samples were incubated at room temperature for 15 min. RNase T1 treatment was performed by adding 1 µl of 0.2 U/µl RNase T1 and the samples were incubated for 15 min at room temperature. Reactions were stopped by adding 10 µl of denaturing formamide loading dye. Alkaline ladder was obtained by incubating 0.1 pmol of labeled *ppa in vitro*-synthesized RNA at 95 °C for 5 min in 7.5 µl of alkaline hydrolysis buffer containing 1.5 µg of yeast RNA. Reactions were stopped by adding 10 µl of denaturing formamide loading buffer. RNase T1 G ladders was obtained by incubating 0.1 pmol of labeled *ppa in vitro*-synthesized RNA and 1 µl of 1 µg/µl yeast RNA in 9 µl sequencing buffer for 10 min at 50 °C, followed by the addition of 1 µl of 0.2 U/µl RNase T1 and incubation at room temperature for 15 min. Reactions were stopped by adding 12 µl of denaturing formamide loading dye. The samples were separated on 8.3 M urea-10% polyacrylamide sequencing gels, and the vacuum-dried gels were exposed to imaging plates. Signals were visualized using a Thyphoon FLA 9500 instrument (GE Healthcare) with Quantity One software (Bio-Rad).

### Library preparation and data analysis

Libraries were prepared following the whole transcriptome protocol as previously described^18^. In brief, 2 µg of affinity-purified RNA samples and 4 µg of control RNA samples (unbound cell lysate of empty beads) were treated with 4 units of TURBO DNase (Life Technologies) in 2 consecutive incubation steps, each at 37 °C for 15 min. Next, RNA clean-up and separation into small (17–200 nt) and large ( > 200 nt) RNA fractions was performed using RNA Clean & Concentrator columns (Zymo Research). The large RNA fraction was fragmented according to the protocol^18^ and was recombined with the small RNA fraction after RNA Clean & Concentrator column purification (Zymo Research). All subsequent follow-up steps were performed according to the protocol^18^ with the following modifications: the clean-up of RNA 5′-pholyphosphatase (Epicentre)-treated samples was performed using Clean & Concentrator column purification (Zymo Research). For RNA adapter ligation, the UGA linker (Supplementary Table S4) was used. After RNA adapter ligation and cDNA synthesis, the samples were gel excised from 2% agarose gels and purified using a NucleoSpin Gel and PCR Clean-up kit (Macherey-Nagel) with the optional NTC buffer to solubilize the gel slices. After PCR amplification, residual primers were removed by adding 10 µl of ExoSAP-IT (USB) to 50 µl of PCR, and with samples incubated for 15 min at 37 °C followed by heat inactivation of the enzyme for 15 min at 85 °C. The samples were cleaned-up using a NucleoSpin Gel and PCR Clean-up kit (Macherey-Nagel), and the cDNA libraries were analysed on an Illumina HiSeq 2500 or 3000 sequencer. Sequencing data were analysed using the Galaxy^43^ Platform https://usegalaxy.eu/. Reads were mapped to the *Prochlorococcus* MED4 genome with BWA-MEM (Galaxy Version 0.7.17.1), unmapped reads were removed with BAM filter (Galaxy Version 0.5.9) and that data was converted to the wig format using BAM to Wiggle (Galaxy Version 2.6.4). To merge different wig files, missing nucleotide positions were added and the read number was set 0. Read numbers were normalized to library size and merged grp files for each strand were generated. Grp files are available as supplementary files 1 and 2.

### Peak calling

The start and end of a peak were set if the difference between the average read coverage over a defined base range separated by a spacer exceeded the threshold (Fig. S2A). In addition, the coverage between the defined start and endpoints had to pass the coverage threshold. In this study, we compared the coverage over 5 nucleotides separated by 10 nucleotides, which had to exhibit a fold change of 1.5 to be considered as the start- or endpoint. The coverage of a peak had to be at least 20% of the average coverage of a nucleotide in the dataset. A schematic overview on the peak calling procedure is presented in Fig. S2A. The input for peak calling was a grp file and the Python script for data analysis is available at https://github.com/SJLambrecht/GRP_Peakcall.

### RNase E cleavage assays and RNase E purification

RNase E purification and cleavage assays were essentially performed as previously described^42^. Yfr2 transcripts (25 pmol) labelled with Cy3 and equal amounts of unlabelled Yfr2 D1, Yfr1, or Yfr10 were incubated with RNase E. Aliquots were withdrawn immediately after reaction assembly and after 20 min of incubation at 30 °C. Prior to analysis of RNA cleavage on 10% urea PAGE, cleavage reactions were purified using an RNA Clean and Concentrator Kit (Zymo).

### Construction of plasmids and GFP reporter assay

To introduce a second sRNA gene (*yfr1*) or the control RNA under its own P_LlacO_ promoter in the pZE12-luc*-yfr2* plasmid, PCRs were performed with primers PromFw-AvrII and Yfr1PromRv-AvrII or primers PromFw-AvrII and pJV300PromRv-AvrII (Table S4) using the pZE12-luc*-yfr2* or pJV300 control plasmid, respectively, as template. The PCR products, pZE12-luc*-yfr2* and pJV300 were digested with *AvrII*, and the amplified *yfr1* gene with the P_LlacO_ promoter was introduced into pZE12-luc*-yfr2* and pJV300. For higher GFP signals, the 5′ UTR of PMM1121 was generated by annealing primers PMM1121_aqua_sense and PMM1121_aqua_as (Table S4). The backbone of pXG10 (expressing sfGFP) was amplified by inverted PCR using the primers pXG10_sfGFP_aqua_righ and pXG10_sfGFP_aqua_left (Tables S4) and a plasmid containing the 5′ UTR of PMM1121 fused to sfGFP was generated by AQUA cloning^44^.

In general, GFP assays were performed as previously described^11^. Briefly, *E*. *coli* Top10 cells were transformed with the plasmids encoding the PMM1121 UTR fused to sfGFP and one of the sRNA encoding plasmids. The colonies were inoculated into 200 µl antibiotics-containing LB medium and grown overnight at 37 °C in a 96-well plate. Cells were diluted 1:10 into fresh LB medium and fixed in 1% HistoFix (Roth). Single-cell fluorescence was determined by flow cytometry using an Accuri C6 flow cytometer (BD Bioscience). Cell fluorescence was measured with an excitation wavelength of 488 nm and the emission was detected at 533/30 nm. The mean fluorescence per plasmid combination was calculated from 50,000 events (cells) of 12 individual clones.

## Supplementary information

Supplementary Information

Supplementary Information

Supplementary Information

Supplementary Information

Supplementary Information

Supplementary Information

Supplementary Information

## Data Availability

The sequencing data have been deposited in NCBI-SRA under accession numbers SRR8280994, SRR8280995, SRR8280996, SRR8280997, SRR8280998 and SRR8280999.
