# Supplementary material for: Interplay and Targetome of the Two Conserved Cyanobacterial sRNAs Yfr1 and Yfr2 in Prochlorococcus MED4
Source: Sci Rep. 2019 Oct 4;9:14331. doi: 10.1038/s41598-019-49881-9 (PMC6778093; doi:10.1038/s41598-019-49881-9)
Supplement: Supplementary file 1 — Supplementary Information [file 41598_2019_49881_MOESM1_ESM.pdf]

Interplay and Targetome of the Two Conserved Cyanobacterial sRNAs Yfr1 and Yfr2 in *Prochlorococcus* MED4

S. Joke Lambrecht[1], Yu Kanesaki[2,3], Janina Fuss[4], Bruno Huettel[4], Richard Reinhardt[4] and Claudia Steglich[1]*

Supplementary Tables, Figures and Files

Table S1. Information on Yfr2 targets enriched after Yfr2 affinity purification. The enrichment factor was calculated by dividing the read coverage of the peak area of the enriched library with that of the control library. Only peaks with an enrichment factor of >2 in both replicates were considered.

Table S2. Information on Yfr1 targets enriched after Yfr1 affinity purification. The enrichment factor was calculated by dividing the read coverage of the peak area of the enriched library with that of the control library. Only peaks with an enrichment factor of >2 were considered.

Table S3. Information on Yfr10 targets enriched after Yfr10 affinity purification. The enrichment factor was calculated by dividing the read coverage of the peak area of the enriched library with that of the control library. Only peaks with an enrichment factor of >2 were considered.

Table S4. List of oligonucleotides used in this study.

Figure S1. Visualization of the CRAFD-Seq workflow. *In vitro*-transcribed sRNAs were biotinylated at their 3' ends and loaded onto streptavidin-coated magnetic beads. Charged beads were incubated with cell lysate, and the bound RNA was processed to generate NGS sequencing libraries followed by sequencing.

Figure S2. Graphical explanation of the computational workflow. A) Definition of peaks. If the average coverage of peak start interval 2 relative to peak start interval 1 exceeded the threshold it was defined as start, with the endpoint detected in a similar manner. In the last step, the coverage between the start and end points had to pass the coverage threshold. B) Details of the annotation and classification of peaks. If the start point of a peak was located in the 5' region of a CDS and the endpoint was within the CDS, the peak was classified as a

5'UTR. If both the start and end points were located inside a CDS, the peak was classified as a CDS. If the start point of a peak was located within the CDS and the endpoint of the CDS was within the 3' region, the peak was classified as a 3'UTR. If the peak was located on the antisense strand with respect to the CDS, the peak was classified as as5'UTR, asCDS or as3'UTR. If the start and end points of a peak were located outside of a CDS, the peak was classified as an IGR.

Figure S3. Left side: RNase E cleavage assays of 12.5 pmol *in vitro*-transcribed Yfr1, Yfr2, Yfr10, or Yfr2 D1 RNAs after 0 or 10 min of incubation with recombinant *Prochlorococcus* RNase E. Cleavage fragments were separated on 7 M urea-6% PAA gels and stained with ethidium bromide or were visualized by detection of fluorescence of Cy3-labelled *in vitro* RNA. The black arrow indicates the full-length transcript and the grey arrow indicates the cleavage product. Right side: Predicted structures of Yfr2 and Yfr2 D1. The RNase E cleavage region in Yfr2 is highlighted in yellow. Nucleotides in cyan result from T7 polymerase-driven transcription.

Figure S4. Box plot of INTARNA prediction rank distribution for Yfr1, Yfr10 and Yfr2 enriched and non-enriched peaks. Statistical significant differences of rank distributions were calculated in Origin 9.1 using the non-parametric Kruskal-Wallis ANOVA test.

Figure S5. Comparison of the lists of genes identified by Yfr1 and Yfr10 affinity purification. The Venn diagram represents the relationship of common and specific targets of Yfr1 and Yfr10.

Figure S6. Identification of new Yfr10 homologs in *Prochlorococcus* SS120 and *Synechococcus* WH8102. Northern blot analysis of samples taken after 0, 24, and 48 h of nitrogen depletion show a small gradual decrease in both *Prochlorococcus* SS120 and *Synechococcus* WH8102 Yfr10 abundance.

Figure S7. Yfr10 homologs in *Prochlorococcus* strains MED4, MIT9313 and SS120, *Synechococcus* WH8102, *Synechocystis* sp. PCC 6803 and *Nostoc* sp. PCC 7120. A) Phylogenetic relationships between strains were calculated by Bayesian interference based on a 16S rRNA alignment. The numbers at the branches indicate the posterior probability. Secondary structures of the Yfr10 homologs were calculated by RNAfold. The black scale bar shows the substitutions per site of the phylogeny, and the coloured scale bar shows the base

pair probability of the secondary structures. B) The alignment of the Yfr10 homologs was calculated using MUSCLE (Edgar 2004, Nucleic Acids Res. 32, 1792–1797) and was visualized in Jalview (Waterhouse et al. 2009, Bioinformatics 25, 1189–1191).

File S1. grp-file of the read distribution for the control library (first track), the Yfr1- (second track), the Yfr2- (third track) and the Yfr10 affinity purified libraries (fourth track) on the forward strand. Read values were normalized to library size. Data can be imported in the Artemis viewer.

File S2. grp-file of the read distribution for the control library (first track), the Yfr1- (second track), the Yfr2- (third track) and the Yfr10 affinity purified libraries (fourth track) on the reverse strand. Read values were normalized to library size. Data can be imported in the Artemis viewer.

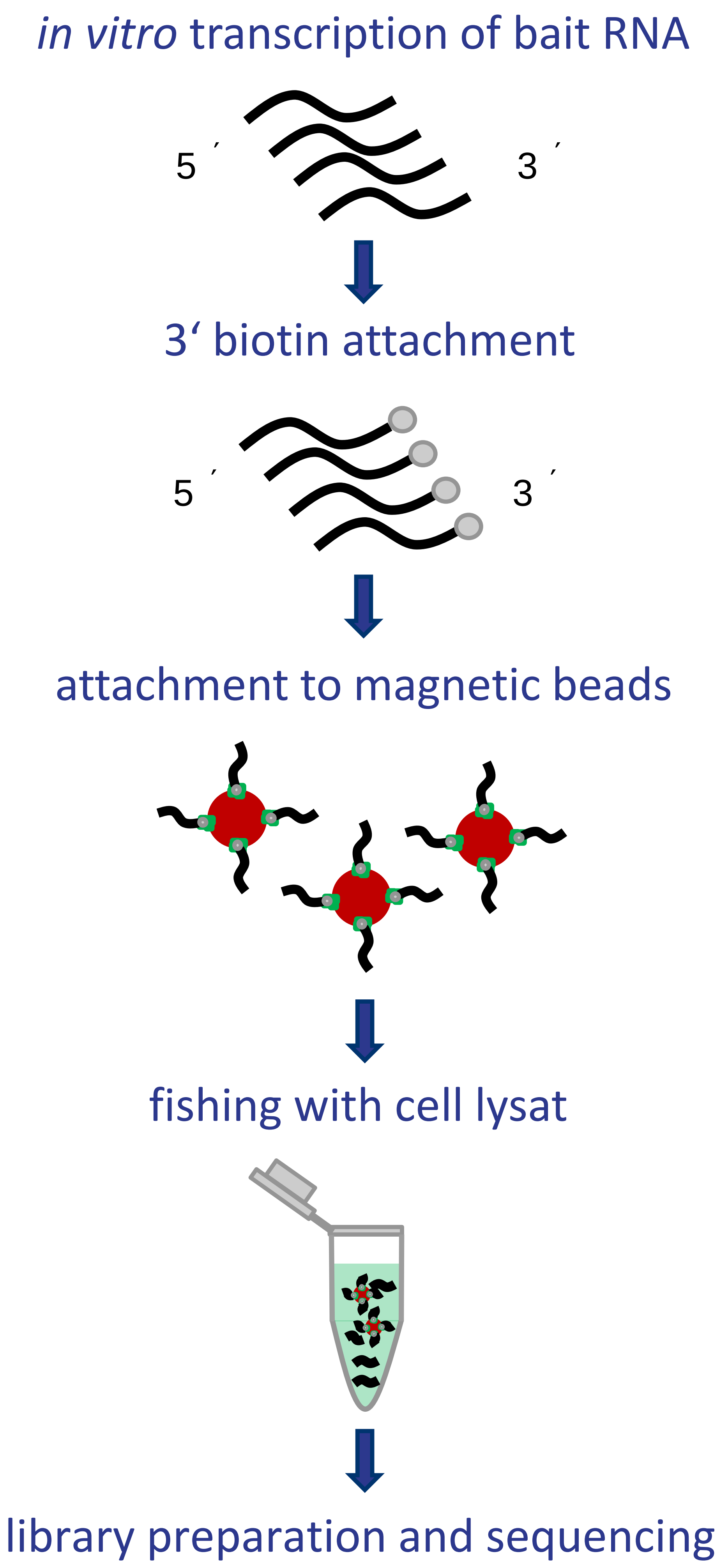

in vitro transcription of bait RNA
5´
3´
3‘ biotin attachment
5´
3´
attachment to magnetic beads
fishing with cell lysat
library preparation and sequencing

Figure S1

A

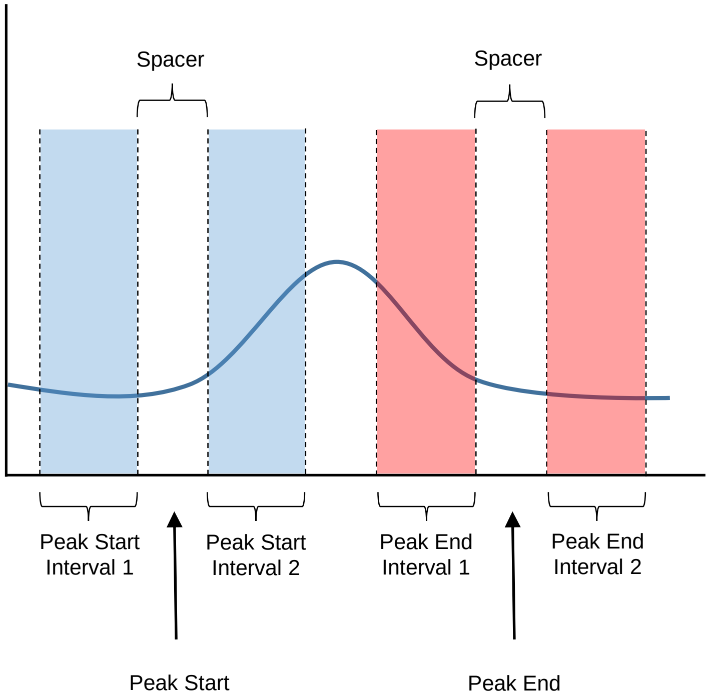

Spacer
Spacer
Peak Start
Interval 1
Peak Start
Interval 2
Peak End
Interval 1
Peak End
Interval 2
Peak Start
Peak End

B

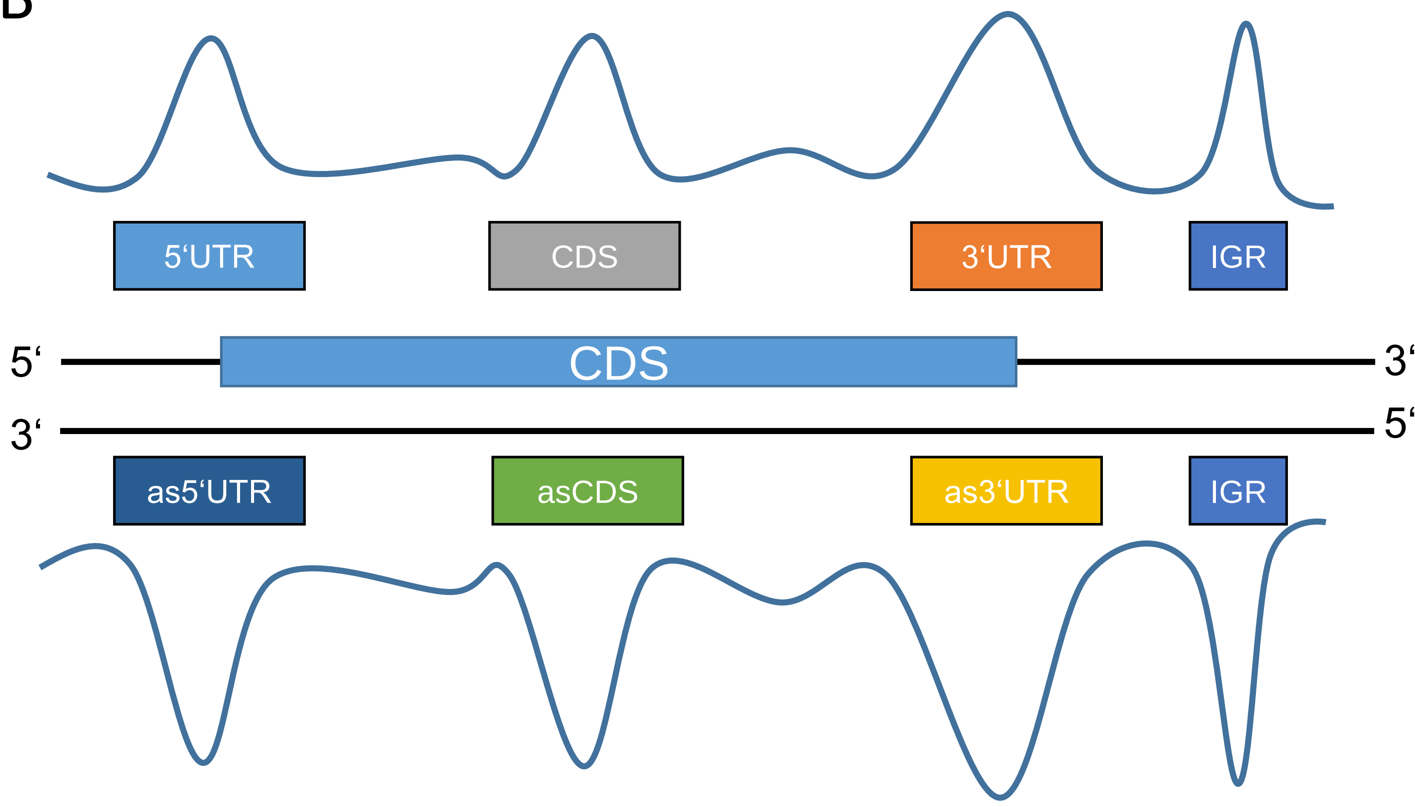

5'UTR
CDS
3'UTR
IGR
5'
CDS
3'
3'
5'
as5'UTR
asCDS
as3'UTR
IGR

Figure S2

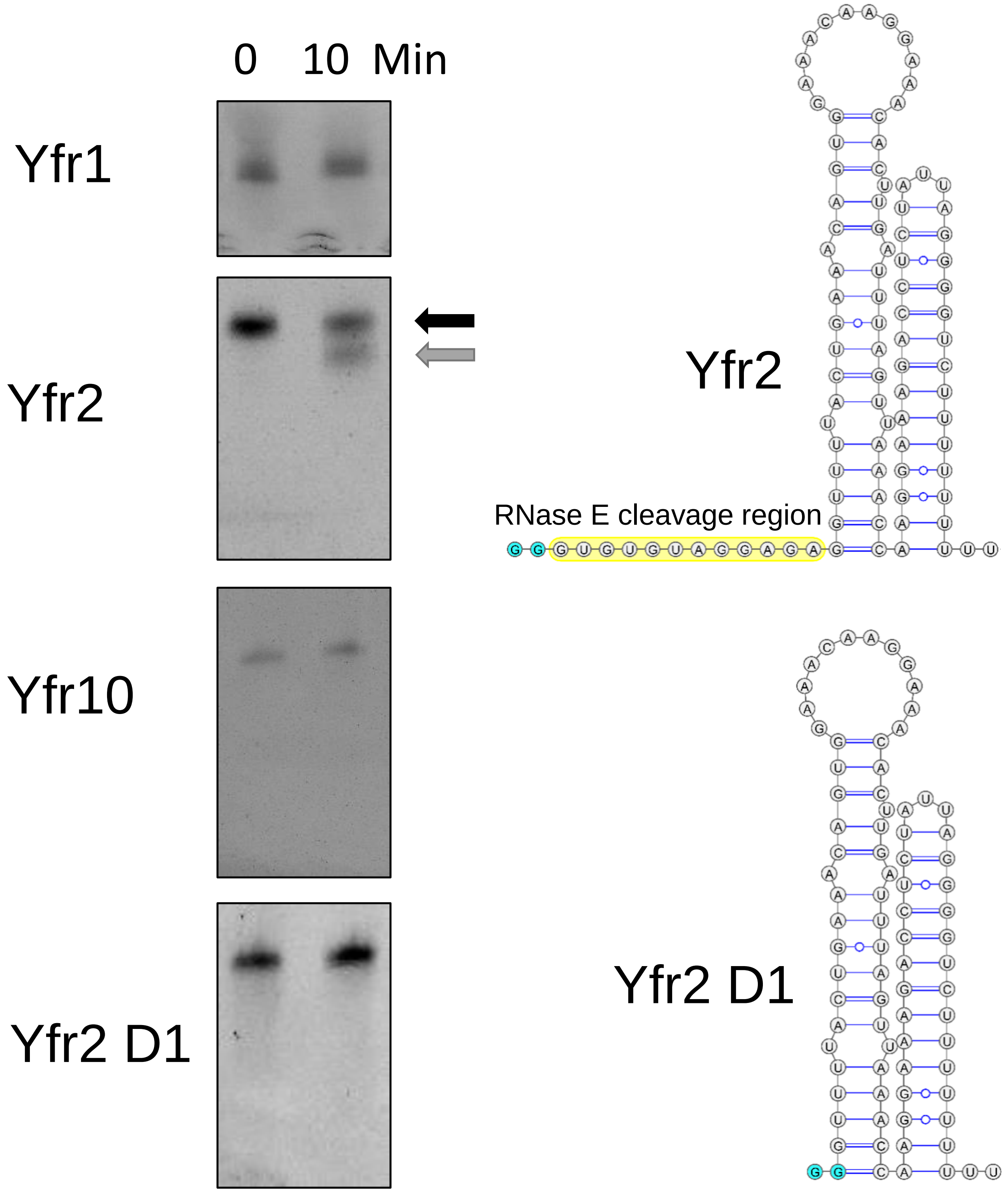
0 10 Min
Yfr1
Yfr2
Yfr10
Yfr2 D1
Yfr2
RNase E cleavage region
Yfr2 D1

Figure S3

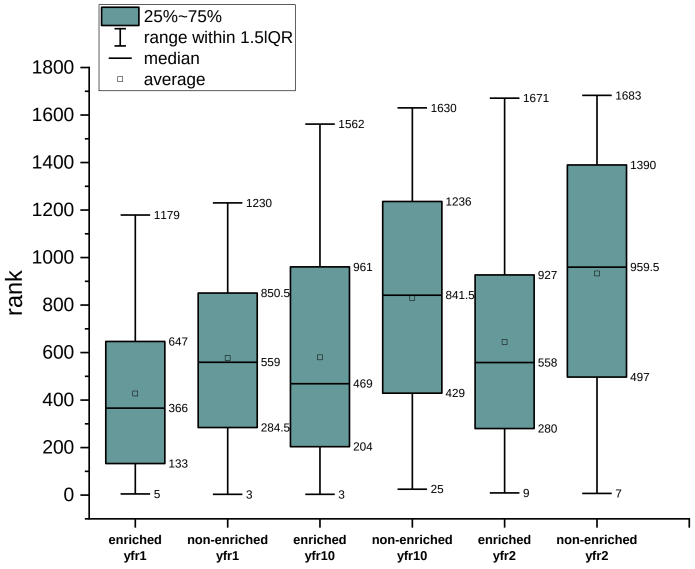
25%~75%
range within 1.5IQR
median
average
rank
1800
1600
1400
1200
1000
800
600
400
200
0
1179
647
366
133
5
1230
850.5
559
284.5
3
1562
961
469
204
3
1630
1236
841.5
429
25
1671
927
558
280
9
1683
1390
959.5
497
7
enriched yfr1
non-enriched yfr1
enriched yfr10
non-enriched yfr10
enriched yfr2
non-enriched yfr2

Figure S4

Number of specific and common targets of Yfr1 and Yfr10

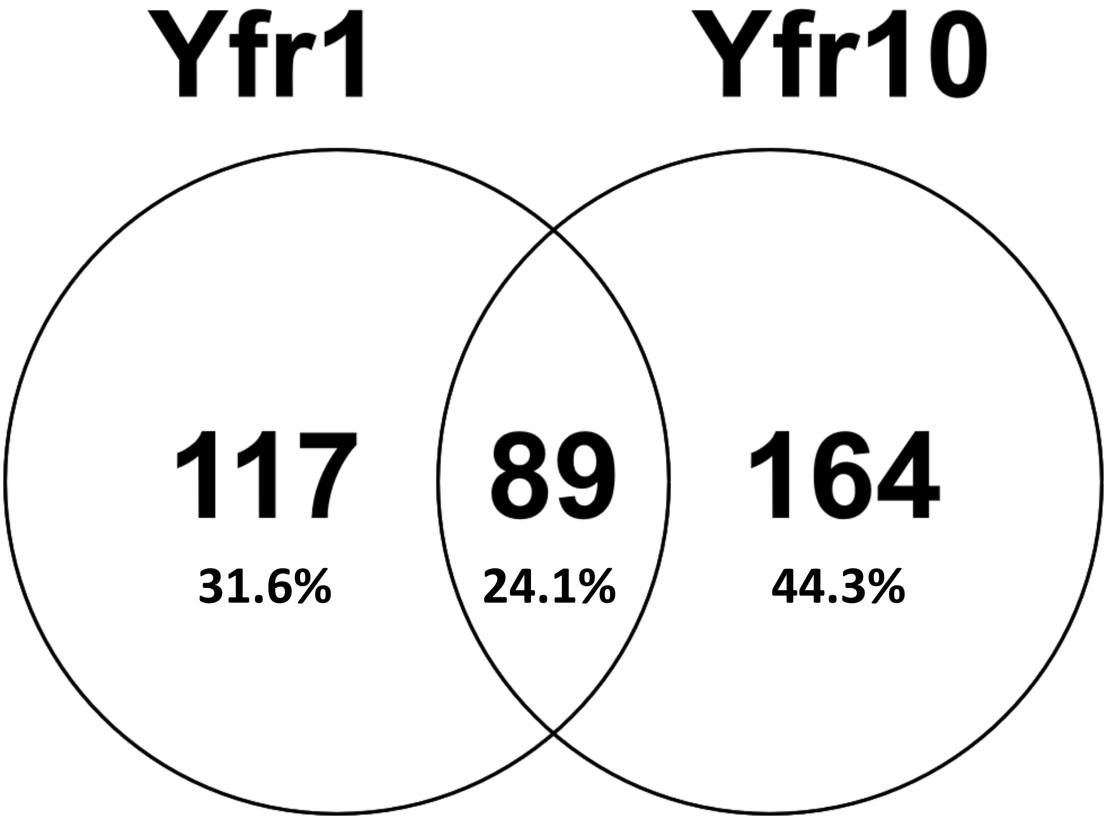

Figure S5

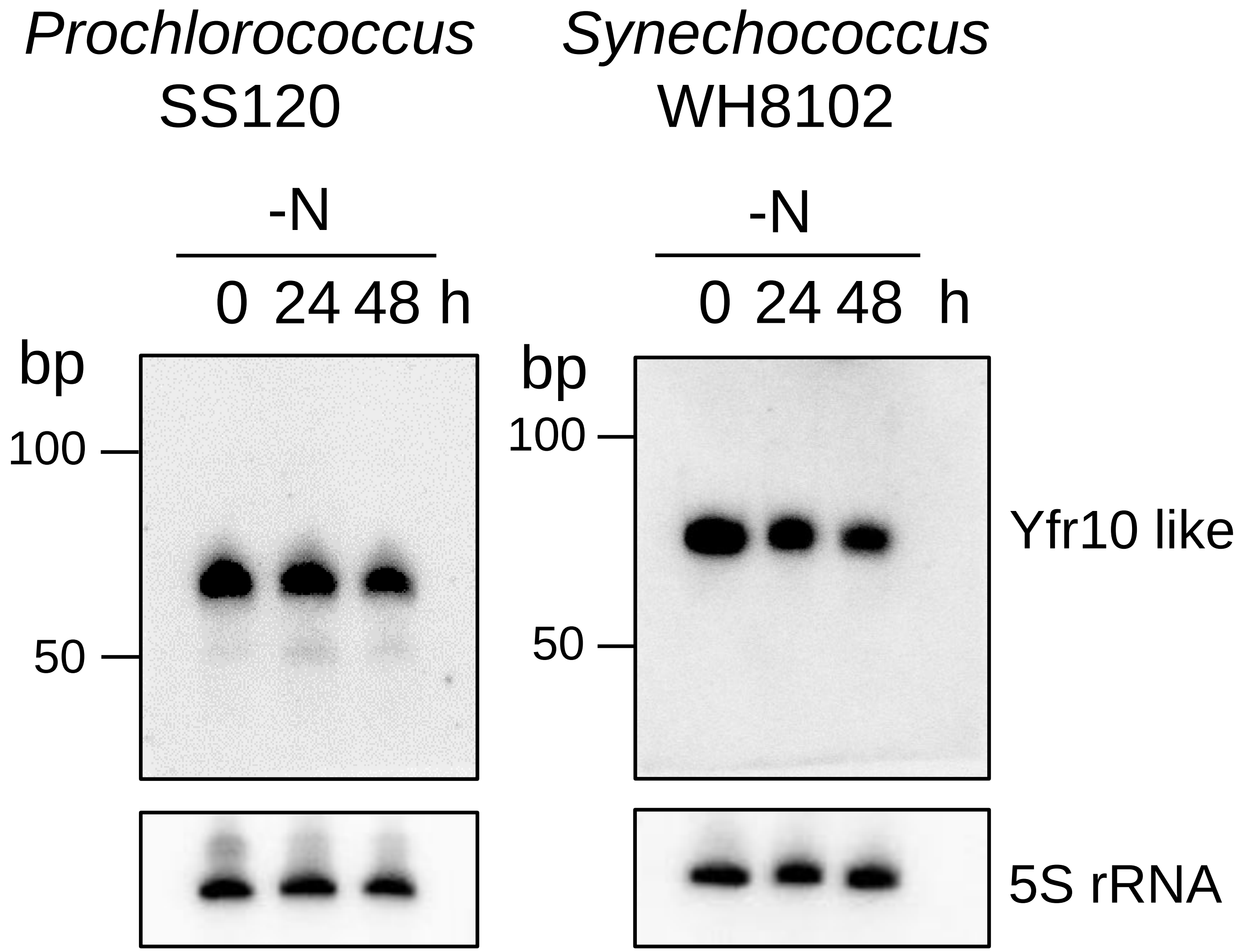

Prochlorococcus
SS120
Synechococcus
WH8102
-N
-N
0 24 48 h
0 24 48 h
bp
bp
100
50
100
50
Yfr10 like
5S rRNA

Figure S6

A

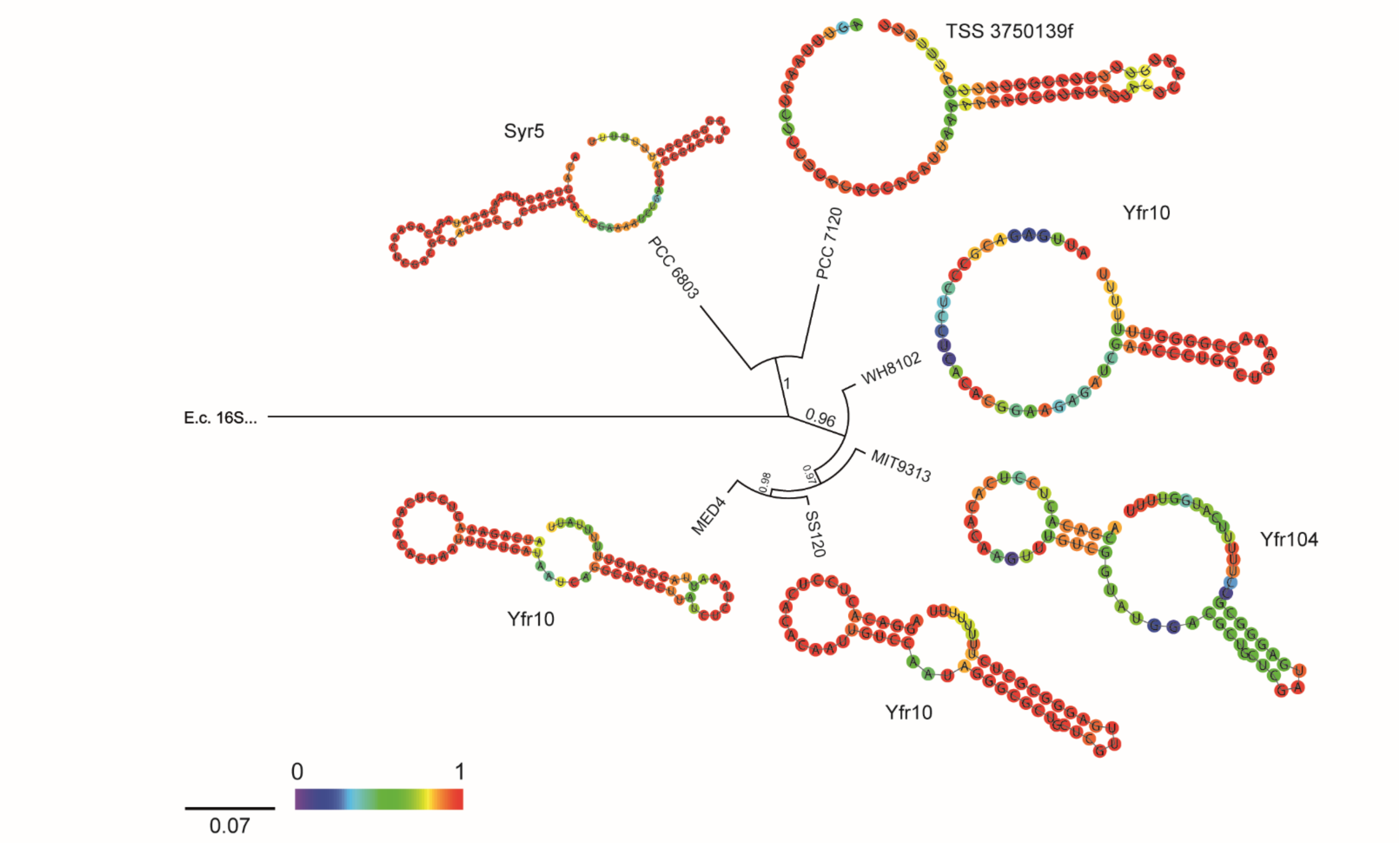
TSS 3750139f
Syr5
Yfr10
Yfr10
Yfr104
Yfr10
PCC 6803
PCC 7120
WH8102
MIT9313
MED4
SS120
E.c. 16S...
1
0.96
0.98
0.97
0
1
0.07

B

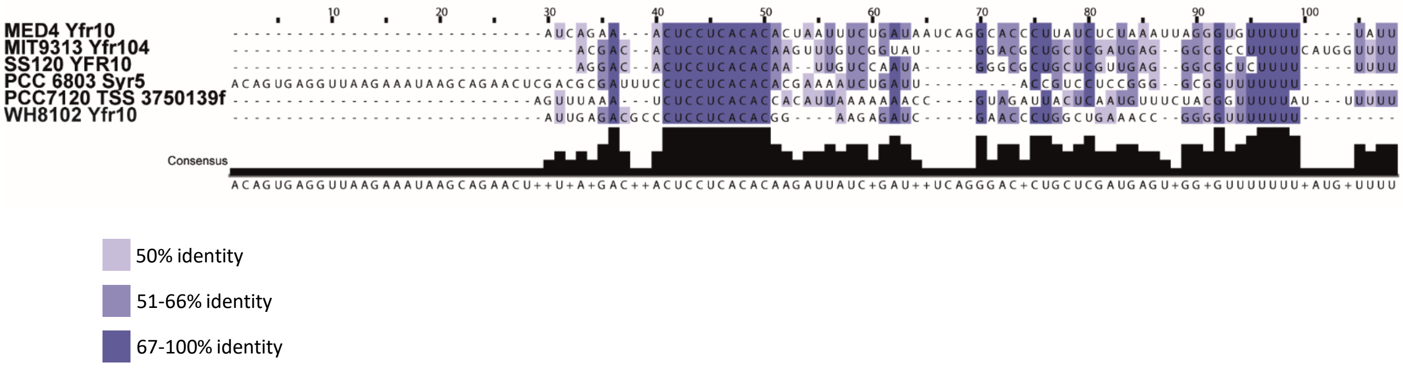
MED4 Yfr10
MIT9313 Yfr104
SS120 YFR10
PCC 6803 Syr5
PCC7120 TSS 3750139f
WH8102 Yfr10
Consensus
ACAGUGAGGUUAAGAAAUAAGCAGAACU++U+A+GAC++ACUCCUCACACAAGAUUAUC+GAU++UCAGGGAC+CUGCUCGAUGAGU+GG+GUUUUUUU+AUG+UUUU
50% identity
51-66% identity
67-100% identity

Figure S7
